# Multiple stressors interact primarily through antagonism to drive changes in the coral microbiome

**DOI:** 10.1038/s41598-019-43274-8

**Published:** 2019-05-02

**Authors:** Rebecca L. Maher, Mallory M. Rice, Ryan McMinds, Deron E. Burkepile, Rebecca Vega Thurber

**Affiliations:** 1 Department of Microbiology, Oregon State University, 226 Nash Hall, Corvallis, OR 97331 USA; 2 Department of Ecology, Evolution and Marine Biology, University of California Santa Barbara, Santa Barbara, CA 93106-9610 USA; 3 Marine Science Institute, University of California Santa Barbara, Santa Barbara, CA 93106-9610 USA; 4 Center of Modeling, Simulation & Interaction, Université Côte d’Azur, Nice, France

**Keywords:** Marine biology, Microbial ecology, Microbiome

## Abstract

Perturbations in natural systems generally are the combination of multiple interactions among individual stressors. However, methods to interpret the effects of interacting stressors remain challenging and are biased to identifying synergies which are prioritized in conservation. Therefore we conducted a multiple stressor experiment (no stress, single, double, triple) on the coral *Pocillopora meandrina* to evaluate how its microbiome changes compositionally with increasing levels of perturbation. We found that effects of nutrient enrichment, simulated predation, and increased temperature are antagonistic, rather than synergistic or additive, for a variety of microbial community diversity measures. Importantly, high temperature and scarring alone had the greatest effect on changing microbial community composition and diversity. Using differential abundance analysis, we found that the main effects of stressors increased the abundance of opportunistic taxa, and two-way interactions among stressors acted antagonistically on this increase, while three-way interactions acted synergistically. These data suggest that: (1) multiple statistical analyses should be conducted for a complete assessment of microbial community dynamics, (2) for some statistical metrics multiple stressors do not necessarily increase the disruption of microbiomes over single stressors in this coral species, and (3) the observed stressor-induced community dysbiosis is characterized by a proliferation of opportunists rather than a depletion of a proposed coral symbiont of the genus *Endozoicomonas*.

## Introduction

In natural systems, disturbances or stressors rarely occur in isolation. Anthropogenic impacts disrupt individual animal physiology, alter whole populations or community dynamics, and drive shifts in system-level processes thereby putting biodiversity in peril^1–4^. Therefore, it is imperative to characterize how multiple stressors interact to disrupt natural systems. We are using the operational definitions of types of interactions between multiple stressors as defined by Folt *et al*.^3^ and Vinebrooke *et al*.^4^. An additive effect, or null interaction, occurs when the combined effect equals the sum of the separate effects. A synergistic interaction occurs when the combined effect of multiple stressors is greater than the additive effect. And lastly, an interaction is deemed antagonistic when combined stressors produce a biological response that is less than the additive effect.

Despite the existence of multiple interaction outcomes, synergies are often emphasized in conservation literature, perhaps due to the risk of negative feedbacks accelerating ecosystem decline and degradation^5^. A balanced research agenda that looks for synergies and antagonisms is necessary to fully understand how mitigating local stressors will or will not compensate for global stressors^6^. For instance, improving water quality and decreasing water turbidity in seagrass systems may exacerbate the damaging effects of heat stress from global warming^6^. Similarly, marine invertebrates and their microbiomes are often faced with global stressors associated with climate change and local stressors such as nutrient pollution or overfishing^7–9^. Yet few studies empirically test the individual and combinatorial effects of more than two stressors on host microbiomes^7–10^.

Current statistical methods and models for microbiome studies^11^, such as those that evaluate alpha and beta diversity and differential abundance, can be combined with multi-stressor experimental designs and used to statistically quantify the interacting effects of multiple stressors. For instance, patients with Crohn’s, a disease associated with gut microbiome dysbiosis, were treated with either antibiotics or a diet of exclusive enteral nutrition^12^. The two therapies likely disrupt the gut microbiome through different mechanisms and are independently associated with dysbiosis. In one case, the stressors produced opposite responses in the abundance of a single bacterial genus, *Alistipes*. Yet, an antagonistic interaction was not tested for but easily could be with a crossed design with patients receiving both therapies. In an environmental example, warm- or cold-stressed oysters crossed with bacterial infection by vibrios showed evidence of synergy as warm-stressed oysters experienced the highest mortality following infection^13^. When evaluating the oyster hemolymph microbiome, an interaction term of stress × infection in the univariate analysis of alpha diversity and multivariate analysis of beta diversity was not included, but if included in statistical methods, would clarify the type of interaction between the two stressors.

Using robust statistical methods and interaction models benchmarked in the microbiome field^14^, we investigated how a global stressor, thermal stress, interacts with local stressors, nutrient pollution and predation, to alter the coral microbiome. Corals, currently experiencing major threats of climate change and nutrient pollution, can function as environmental sentinels and are thereby prime candidates for multiple stressor experiments. Previous studies of the coral microbiome have shown that stress tends to increase species richness^8,9,15–17^ and cause shifts in community composition from potentially beneficial symbiotic bacteria that dominate healthy corals to potentially opportunistic or pathogenic bacteria that dominate stressed corals^8,9,15,17–20^. Beta diversity, or species turnover between samples, has also been reported to increase with stress^7,9,21,22^, and stressed corals have microbial communities distinct from control corals^23–26^.

Therefore, we designed a fully-crossed experiment to investigate biological responses including alpha and beta diversity indices and differential abundance modeling of individual taxa with stress. For the purposes of this study, we define a stressor to be any external disturbance from the host’s environment that causes a quantifiable change in microbial community structure. We utilized univariate and multivariate statistical techniques to parse the main effects and interactions among stressors. The coral *Pocillopora meandrina* was exposed to increased seawater temperatures, pulse nitrate and ammonium enrichment, and simulated predation in a factorial mesocosm tank experiment with all possible combinations of these stressors. We hypothesized that local stressors like nutrient pollution and predation would interact synergistically with thermal stress to reduce the host’s ability to regulate its microbial community which would be manifested by: (1) an increase in the compositional heterogeneity and variability (beta diversity) among stressed corals compared to the controls, and (2) an increase in community evenness in stressed corals as a result of (3) shifts from few dominant symbiotic bacterial taxa to a myriad of potentially opportunistic bacterial taxa that bloom and become overrepresented in stressed corals.

## Results

### Stressors drive symbiont decreases and opportunist increases in relative abundance

**Figure 1:**
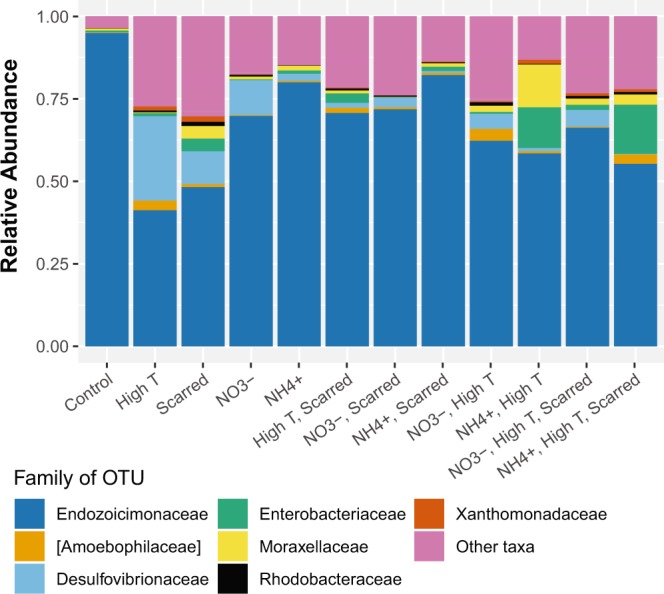
Distribution of the relative abundance of dominant OTUs across the twelve experimental treatments from the rarefied OTU table. The top nine OTUs with a mean relative abundance >0.005 are plotted and colored by family. Three of the top OTUs belong to the family *Moraxellaceae*. All other family labels represent a single OTU. All other OTUs are grouped in “Other taxa” to visualize 100% of the community.

To test the individual and interactive effects of increased temperature, nutrient enrichment, and predation on coral microbiomes, replicates of the coral *Pocillopora meandrina* were exposed to each combination of individual, double, and triple stressors in a fully crossed tank experiment (Supplementary Fig. S1). Patterns in the relative abundance of different microbial taxa were assessed with generalized linear mixed-effects models (GLMM) and clearly revealed a dominant member of the coral community (Fig. 1). A single OTU assigned according to the Greengenes database to the family *Endozoicimonaceae* (OTU-Endo, genus *Endozoicomonas*) was present in every sample unit with relative abundances ranging from 0.093–99.44%, with an average 67.52 ± 3.55%. Members of the genus *Endozoicomonas* are proposed bacterial symbionts with coral, and are typically underrepresented in stressed corals^8,27–29^. All control corals were dominated by OTU-Endo with an average relative abundance of 94.88 ± 0.86% (Fig. 1). The main effects of high temperature, scarring, and NO_3_^−^ all significantly decreased the relative abundance of OTU-Endo in the GLMM (p < 0.001, p < 0.001, p < 0.05; Supplementary Table S1, Fig. 1). Compared to control corals, the relative abundance of the dominant OTU-Endo decreased by more than 50% in the high temperature treatment (41.19 ± 16.61%) and decreased nearly 50% in the scarred treatment (48.21 ± 16.61%). High temperature and scarring interact to reduce the sum of the independent effects on the dominant taxon (p < 0.01; Supplementary Table S1), decreasing OTU-Endo by only ~25% (70.67 ± 5.44) compared to controls. In fact, all two-way interactions were antagonistic and significantly reduced the magnitude of the response predicted by main effects (Supplementary Table S1). Alternatively, both three-way interactions significantly increased the response of the individual stressors on OTU-Endo after accounting for all main effects and two-way interactions (Supplementary Table S1).

The decreases observed in OTU-Endo closely mirror increases in the second most abundant OTU of the family *Desulfovibrionaceae* (OTU-Desulfo). For instance, all four main effects significantly increased the relative abundance of OTU-Desulfo (Supplementary Table S2) with high temperature causing the greatest change in this OTU from control corals with 0.09 ± 0.09% to 25.56 ± 13.44%, followed by NO_3_^−^ (10.62 ± 10.18%) and scarring (9.92 ± 6.30%). Alternatively, the interaction of high temperature and scarring only increased the relative abundance of OTU-Desulfo to 1.51 ± 0.78%. Two-way and three-way interactions showed similar significant, but opposite directional changes as those observed for OTU-Endo (Supplementary Table S2). For the third most abundant OTU of the family *Enterobacteriaceae*, the main effect of scarring caused significant increases in abundance and significant interactions between scarring and nutrient treatments (Supplementary Table S2). The OTU from family *Amoebophilaceae* significantly increased in scarring and high temperature and the three-way interaction with NH_4_^+^ was significant (Supplementary Table S2). And lastly, the OTU from family *Moraxellaceae* significantly increased in the NH_4_^+^ treatment and two-way interactions with scarring and nutrient treatments were significant (Supplementary Table S2). For all OTUs, significant two-way interactions were antagonistic, or less than the sum of the main effects, and three-way interactions were synergistic when accounting for main effects and two-way interactions. When evaluating changes in relative abundance, the microbial community under stress is marked both by a decrease in the dominant symbiont and increases in lower abundant opportunistic taxa.

### High temperature and scarring drive changes in community diversity

Alpha diversity metrics were assessed with linear mixed effects models (LMM) and showed the absence of synergisms. On average, Chao1 estimates found that control corals had a mean of 40.42 ± 3.48 unique OTUs and had the lowest standard error across all treatments (Supplementary Fig. S2). Neither main effects nor interactions were significant predictors in the LMM with Chao1 index as the response variable (Supplementary Table S3). Faith’s phylogenetic diversity ranged from 4.09 ± 0.55 in control corals to 8.99 ± 1.81 for corals in high temperature. The main effects of high temperature and scarring significantly increased Faith’s phylogenetic diversity (p < 0.05, p < 0.05; Supplementary Table S3). Additionally, the interaction between high temperature and NH_4_^+^ showed a significantly antagonistic response on Faith’s phylogenetic diversity (p < 0.05, Supplementary Table S3). Although evidence suggests that stressors generally increase microbial species richness in coral microbiomes^8^, we found no significant differences in species richness, but rather changes in phylogenetic diversity with stressors.

**Figure 2:**
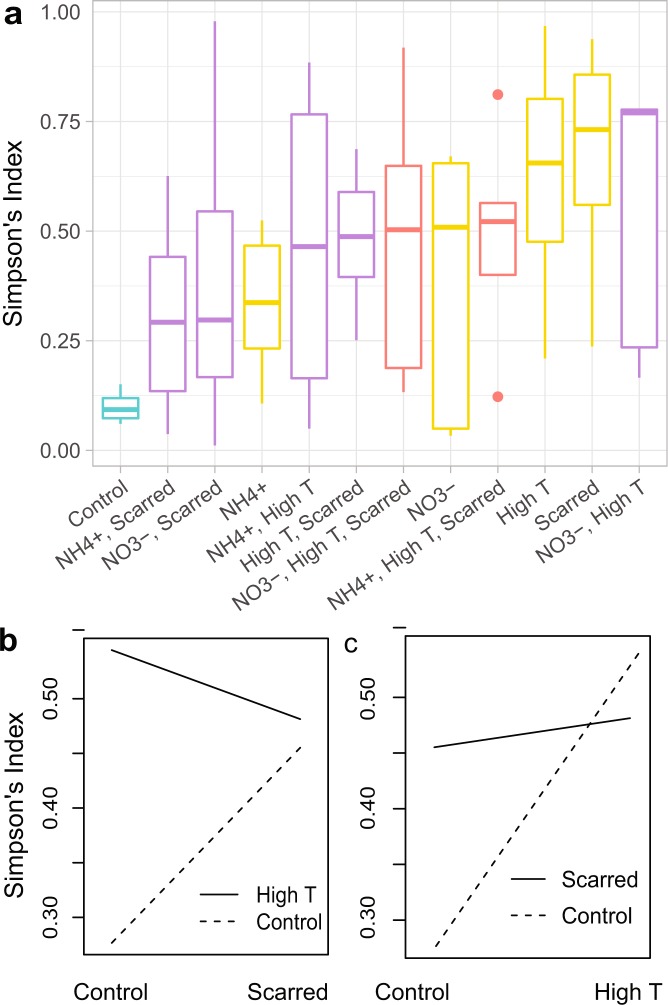
Patterns in community alpha diversity. (**a**) Average Simpson’s index by treatment. Box colors represent the type of stressor combination applied to the corals: none = teal, single = yellow, double = purple, triple = red. No pairwise treatment comparisons were significant, however, several main effects and interactions were significant in the linear mixed effects model (Supplementary Table S3), including the interaction between scarring and temperature. (**b**,**c**) Interaction plots of interaction between scarring and temperature on Simpson’s Index visualized two ways.

All treatments tended to increase Simpson’s index or community diversity (3- to 7-fold) compared to the controls, with an index of 0.10 ± 0.02 (Fig. 2a). Patterns in community diversity closely match those of the dominant OTU-Endo and reflect changes in community ‘evenness’ which is accounted for when calculating the Simpson’s index. Both high temperature and scarring significantly increased Simpson’s Index compared to the controls (p < 0.01 and p < 0.01; Supplementary Table S3). High temperature and scarred treatments produced the highest mean community diversity (0.62 ± 0.16 and 0.67 ± 0.11, respectively), greater than 6 times that of the controls. Interestingly, scarring and high temperature interact to reduce the independent effects in the regression analysis (p < 0.01; Supplementary Table S3). This interaction can be observed with interaction plots and described in two ways: (1) high temperature reverses the effect of increased community diversity from control to scarred corals (Fig. 2b), or (2) scarring decreases the difference in community diversity between corals in ambient to high temperature seawater (Fig. 2c). Enrichment with NH_4_^+^ or NO_3_^−^ also significantly interacts to reverse the main effect of scarring, thereby decreasing community diversity in scarred corals compared to controls (p < 0.01, p < 0.05; Supplementary Table S3; Supplementary Fig. S3). The two three-way interactions between scarring, high temperature, and nutrient enrichment also produced a significant result (p < 0.05, p < 0.05; Supplementary Table S3), suggesting that the interaction between any two stressors depends on the level of the third stressor. In the linear model framework, interaction type is not directly interpretable with the less-than- or greater-than-additive definition since three-way interactions in the model account for all main effects and two-way interactions. Therefore, the three-way interaction increased community diversity after accounting for all antagonistic two-way interactions, although the triple stressor treatments are less than most single stressor treatments (Fig. 2a). Despite the significant main effects and interactions in the LMM, no pairwise treatment comparisons were significant with a Tukey post hoc test.

### Beta diversity measures show less-than-additive effects during microbiome exposure to multiple stressors

**Figure 3:**
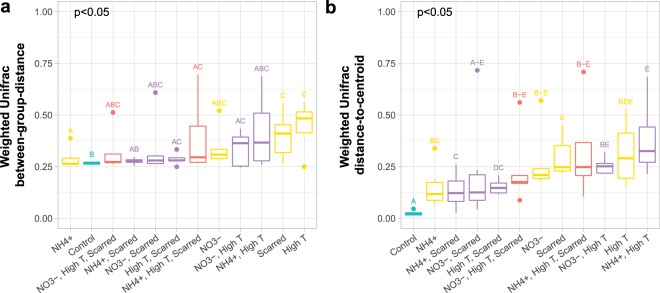
Patterns in community beta diversity. (**a**) Mean between-group distances from Weighted Unifrac community dissimilarity by treatment. P-values denote the significance of treatment group in a PERMANOVA using adonis. Significance codes for each treatment are assigned based on the results of pairwise treatment comparisons with adonis (Supplementary Table S4). (**b**) Weighted Unifrac mean distance-to-centroid by treatment. P-values denote the significance of treatment group in a PERMDISP using betadisper. Pairwise betadisper was used to assign significance codes for group distances (Supplementary Table S6). Groups sharing a letter are not significantly different from each other. Box colors represent the type of stressor combination applied to the corals: none = teal, single = yellow, double = purple, triple = red. Distances are ordered by increasing median, and note that red (triple stressor) treatments are not clustered on the far left.

PERMANOVA results, with treatment as the predicting factor, showed the presence of distinct microbial communities (Fig. 3a p-value, Supplementary Table S4). To visualize differences in community location, mean separation from communities in other treatments (between-group-distance) was calculated (Fig. 3a). Control corals had low average between-group distances and were significantly different from single stressors of NH_4_^+^, high temperature, and scarring (Fig. 3a, Supplementary Table S4). However, unlike our hypothesis that with additional amounts of stress microbiomes would become increasingly distinct, single stressors of scarring and high temperature produced the greatest mean between-group distances rather than interacting stressors (Fig. 3a, yellow versus purple and red boxes). In fact, high temperature and scarring combined was significantly different from controls but not from either high temperature or scarring alone (Fig. 3a, Supplementary Table S4). Additionally, triple stressors (red boxes) were not significantly different from single stressors (Fig. 3a, Supplementary Table S4). Therefore, the combination of multiple stressors had less-than-additive effects on the change in composition of the microbial communities. And, while a true synergism is not possible for relative distance measures with a maximum value of 1.0, combined stressors are generally less than single stressors. The linear model PERMANOVA results showed that in corals experiencing the main effect of high temperature, microbial communities were significantly distinct from controls (p < 0.05; Supplementary Table S5). The three-way interaction between temperature, nutrients, and scarring was also significant (p < 0.05; Supplementary Table S5), suggesting that the biological response to a single stressor is influenced by the other two. Like double stressor treatments, triple stressors (Fig. 3a red boxes) interact antagonistically to produce less community distance or distinctness than scarring or high temperature alone. Our results suggest that the high temperature as a main effect and high temperature and scarring as independent treatments have the most influential effect on shifting microbial communities.

PERMDISP results also showed significant differences among treatments in dispersion magnitude (Fig. 3b p-value, Supplementary Table S6). Distance to group centroid measures showed that control corals harbored microbial communities that were more homogenous, and therefore less dispersed, than those belonging to stressed corals (Fig. 3b). The addition of stressors (with the exception of NO_3_ and scarring combined) increased distance-to-centroid and increased variance suggesting that stress causes dispersion or destabilization of the microbiome. These stochastic changes may show evidence of the Anna Karenina Principle (AKP) in which dysbiotic animal microbiomes vary more in community composition than healthy microbiomes^21^. When considering the dominance of OTU-Endo within the community (Fig. 1), however, evidence suggests that the single taxon is driving the dispersion effect. In fact, the relative abundance of OTU-Endo was significantly negatively correlated with sample distance-to-centroid measurements (estimate: −0.35, p < 0.001). The ability to detect statistically meaningful variance in other taxa is marginal due to the dominance of OTU-Endo. Instead, dispersion may be artificially increased in stressed corals due to an increase in the relativized number of rare taxa and the reduction in OTU-Endo dominance.

### Differential abundance analysis shows stress is marked by an increase in opportunists

**Figure 4:**
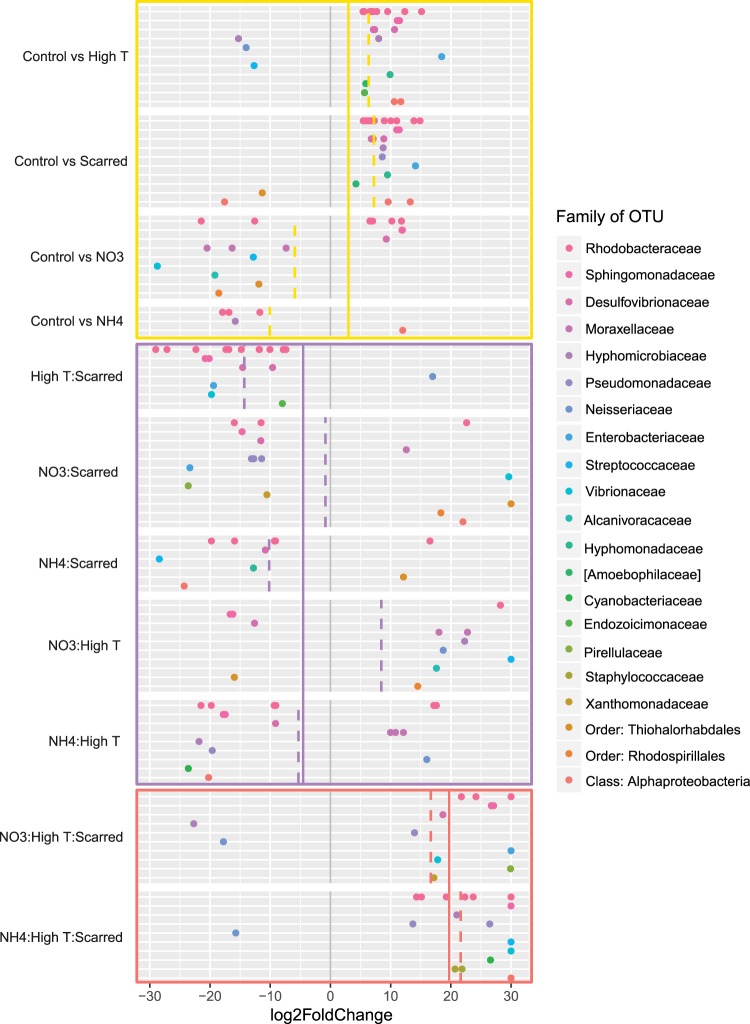
Differential abundance of OTUs with significant response to stressors using DESeq2 and the generalized linear model framework. Only OTUs present in greater than 15 samples were included in the analysis. Each point represents a single OTU that increased or decreased significantly (FDR corrected p < 0.05) with the stressor or stressor interaction. Each row and dot color corresponds to a microbial family (i.e. multiple OTUs from a single family are increased in multiple treatments). Family name in [] represents a recommended taxonomic annotation by GreenGenes. Box colors represent the stressor type: main effect = yellow, two-way interaction = purple, three-way interaction = red. The colored line within each box represents the mean log2FoldChange for OTUs with significant changes in that stressor type. The dashed colored line within comparisons represents the mean log2FoldChange for OTUs with significant changes within the individual stressor effects and interactions. The gray colored line at 0 log2FoldChange denotes no effect or no interaction.

From differential abundance analysis with DESeq2, a total of 56 unique OTUs were differentially abundant in one or more of the treatments or interactions (Fig. 4, Supplementary Table S7). On average, main effects resulted in a 2.80 log2 fold-change in the differentially abundant OTUs (yellow-colored line; Fig. 4). This increase was driven primarily by high temperature and scarred treatments which have an average log2 fold-change of 6.35 and 7.23, respectively (yellow-dashed lines, Fig. 4). In contrast, NO_3_^−^ and NH_4_^+^ treatments on average significantly decreased taxa compared to controls (−5.92 and −10.06, respectively, yellow-dashed line, Fig. 4). The NH_4_^+^ treatment resulted in the fewest differentially abundant OTUs (Fig. 4), suggesting that the microbial communities under NH_4_^+^ enrichment are most similar to the control corals. Ammonium (NH_4_^+^) is a fish-derived form of nitrogen that can be beneficial to corals^30^, which likely would reflect a healthy microbial community. A single OTU from the proposed symbiont family of *Endozoicimonaceae* increased in abundance in high temperature, however, this taxon was not the same dominant OTU-Endo from Fig. 1. In fact, OTU-Endo was not identified as having significantly changed in any treatments. When differential abundance analysis was repeated with an OTU table summarized by Family and Genus, higher-level significant changes generally agree with those of individual OTUs (Supplementary Table S8). Therefore, the average increase in abundance of OTUs from families such as *Rhodobacteraceae*, *Sphingomonadaceae*, and *Desulfovibrionaceae* (Fig. 4), suggests that the changes in relative abundance and dysbiosis resulting from stressors are characterized by an enrichment of pathogenic or opportunistic bacteria rather than a depletion of symbionts.

### Interaction type depends on the type and number of stressors

The response of bacterial taxa in two-way interactions was variable depending on family of OTU, OTU, and type of interacting stressors. Generally, two-way interactions between stressors were antagonistic in nature aside from the interaction between NO_3_^−^ and high temperature which produced a synergistic average increase in taxa (purple-colored box, Fig. 4). In the linear framework model, zero log2-fold change between two main effects (grey lines in purple-colored box, Fig. 4) signifies no interaction or the additive model (sum of the two main effects). Instead, the model shows that the average log2-fold change in bacterial taxa for two-way interactions was less than the sum of the individual main effects by −4.95 (purple-colored line, Fig. 4). This suggests that when combined, two individual stressors act antagonistically to dampen the main effects. The family *Rhodobacteraceae* had the most differentially abundant OTUs. A total of 10 and 14 OTUs from this family significantly increased in abundance in the high temperature and scarred treatments, respectively, whereas, 11 of these shared OTUs decreased in the two-way interaction. Likewise, three OTUs of the family *Desulfovibrionaceae* all increased in the high temperature and scarred treatments. However, in the two-way interaction, two of these OTUs decreased compared to the expected additive model.

The GLM model, however, also shows that three-way interactions between stressors are generally synergistic when considering changes in taxa abundances (red-colored box, Fig. 4). The three-way interaction takes into account the main effects and each of the two-way interactions. The null model predicts that the addition of a third stressor has no effect on the interaction of the other two stressors. Compared to this null model, the three-way interactions of high temperature, scarring, and nutrients resulted in an average 19.55 log2 fold increase in taxa (red-colored line, Fig. 4). Numerous differently abundant OTUs in the two-way and three-way interactions have a 30 log-fold change. These results may, however, be a caveat of the DESeq2 method to calculate a change in abundance associated with the presence of a taxon that was formerly absent in the treatment contrast. Notably, the magnitude of the average log2 fold-change increases with increasing number of stressors (yellow-, purple-, and red-colored lines, Fig. 4), which likely results from reduced power from the consecutive addition of interaction terms, thereby, requiring a larger change for a significant statistical result. Regardless of these differences in the magnitude of the response, the evaluation of factorial interactions with a GLM agree with results from the dominant taxa (Supplementary Tables 1 and 2) and Simpson’s Index (Supplementary Table 3) in characterizing double stressors as antagonistic and triple stressors as synergistic.

## Discussion

**Figure 5:**
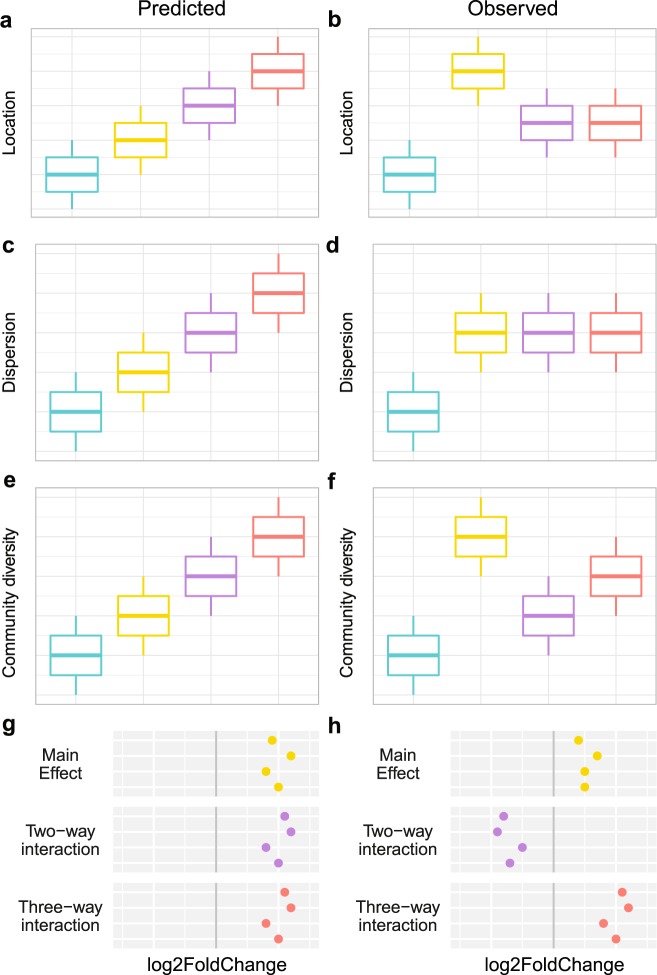
Conceptual description of predicted (**a**,**c**,**e**,**g**) vs observed (**b**,**d**,**f**,**h**) patterns with multiple stressors. Location (**a**,**b**) and dispersion (**c**,**d**) effects represent measures of beta diversity. Community evenness (**e**,**f**) represents patterns in Simpson’s index. Patterns in taxa differential abundance in log2FoldChange using the generalized linear model framework are displayed in g and h with the gray line denoting no effect or no interaction. Colors represent the type of stressor combination applied to the corals: none = teal, single = yellow, double = purple, triple = red.

Contrary to our hypothesis, our overall results suggest the global and local stressors tested in this tank experiment generally do not act synergistically to induce dysbiosis in the coral microbiome of *Pocillopora meandrina*. In fact, we find that the biological response in the microbial community to stress does not scale positively with increasing number of stressors. We predicted that when sequentially adding stressors to the system, we would see a concurrent increase in deterministic changes to the microbiome (Fig. 5a,c,e). For beta diversity, deterministic changes would produce clusters with increasingly distant locations from the control community (Fig. 5a). Stochastic changes would likewise produce communities that were more dispersed or variable (Fig. 5c). Instead, we found that the greatest deterministic changes in the microbial community resulted from single stressors, while interactions produced an intermediate level of change resulting in antagonisms that decreased the individual effects (Fig. 5b). For stochastic changes, any environmental stressor was sufficient to induce dispersion around the centroid of healthy corals (Fig. 5d), although this dispersion was likely a result of the single dominant taxon (Fig. 1). The changes in alpha diversity, however, did not scale positively with the number of stressors, and single stressors appear to increase community diversity more than two or three stressors combined (Fig. 5e,f). We also found that stress induced a myriad of opportunists to invade the community, shifting species dominance away from coral symbionts. The dynamics observed in species’ abundance profiles of the microbial community following a perturbation may be explained by each particular microbes’ nutrient preference and competitive ability^31^.

In contrast to more heterogeneous communities that may be more robust to changes in community evenness, the control corals were dominated by a single taxon initially and thus exhibited low evenness. We would predict then that any perturbation to the system would only increase evenness reflected in higher Simpson’s diversity (e.g., Fig. 2). As such, when stressors were applied to the coral host, bacterial community evenness increased when the dominance shifted from the OTU-Endo symbiont to other taxa such as *Desulfovibrionaceae*^8,19,32^, *Enterobacteraceae*^20,23^, *Amoebophilaceae*^33,34^, *Moraxellaceae*^35,36^, and *Rhodobacteraceae*^8,16,37,38^ (Fig. 1). Contrary to previous work^8^, we did not see an increase in species richness with stress. This may be a result of the mesocosm tanks restricting natural presence/absence dynamics on the reef. Instead, the results suggest a reshuffling of microbial members rather than an increase of new species. Many microbiome studies seek to understand whether dysbiosis, or an imbalance in microbiota, is marked by an invasion or proliferation of pathogens or a depletion of beneficial bacteria^39,40^. Yet taxon relative abundance measures alone do not provide enough information to answer this question.

Individual responses of taxa to stress and their contribution to microbiome dysbiosis can be assessed with differential abundance analysis^37,41,42^. Unlike diversity measures which are driven by the changes of dominant taxa in the community, differential abundance testing can identify changes in minor players in the community. DESeq2 can be used to model the abundance of each taxon independently, while accounting for the discrete positive nature of count data and the compositionality of the community using a generalized linear model (GLM)^43^. Using the linear model framework, we expected main effects to increase opportunistic bacteria, and interactions to produce synergistic effects as the community becomes increasingly compromised (Fig. 5g). Instead, we found that two-way interactions produced antagonistic responses among opportunistic taxa. This apparent antagonism may be a result of a dominance effect, in which one stressor accounts for most or all of the biological response, changing susceptible taxa such that the second stressor has no additional effect^5^. Stressors may provide some benefit or resource that normally limits the abundance of opportunistic taxa. For instance, high temperature may increase bacterial reproduction and metabolic rate, while scarring may increase free nutrients in the form of amino acids or open niches. These results suggest that opportunists such as *Rhodobacter* or *Desulfovibrio* spp. are not co-limited by the resources provided by high temperature and scarring. For instance, opportunistic taxa may be proliferating at such a high rate due to increased temperature and increased reproductive rates, that additional free nutrients from scarring do not compound the effect. In contrast, three-way interactions resulted in synergies as invading taxa continued to increase in abundance (Fig. 5h). This suggests that opportunistic taxa that had maximized their biological response under two stressors, were in fact co-limited by some resource provided by a third stressor. For instance, the addition of nitrogen may have allowed some opportunistic taxa to surpass the maximum threshold of reproductive or metabolic potential set by high temperature and scarring. Alternatively, the difference in interaction type between two-way and three-way interactions may be a result from the coral host’s compromised immune system^44–46^. The coral host effectively regulates it’s associated microbial community under two stressors with a heightened immune response. However, with the addition of a third stressor, innate immunity could be overwhelmed, and the host could no longer regulate its community, thereby allowing a synergistic proliferation of opportunists.

Despite the current bias in interaction literature toward identifying synergies^5^, our study highlights multivariate and univariate statistical tools that can be combined with standard methods in microbial ecology to more accurately characterize interaction types to host-microbiome systems. Community diversity measures are standardly conducted in microbiome research^14^, however, they have rarely been used to explicitly test for antagonisms or synergisms between environmental stressors using a microbiome dataset^7,9,12,13,37^. Although there is no evidence that these measures respond linearly to stress, these analyses revealed unexpected patterns of community response to increasing amounts of stress. This study presents an initial evaluation of the utility of these community diversity measures in characterizing interactions between different combinations of stressors that are known to damage the coral host and produce compositional changes in its microbiome.

## Methods

### Experimental design and sampling of coral tissue for microbial analysis

To test the individual and interactive effects of increased temperature, nutrient enrichment, and predation on coral microbiomes, a fully crossed tank experiment (Supplementary Fig. S1) was conducted at the University of California Gump Research Station (17°29′26.04″S, 149°49′35.10″W) in Mo’orea, French Polynesia. The experiment was conducted in September of 2016 using twelve independent 150 L flow-through, temperature controlled mesocosms with natural locally-sourced sea water at a flow rate of 448.1 ± 24.1 mL per minute and run under a 12:12 light:dark cycle with ~700 μmol quanta per m² per second. Each of the twelve tanks was independently regulated for temperature (chilling loop and heater) and for light. Ten *Pocillopora meandrina* colonies were collected from 3–4 m on the Mo’orea north shore fore reef and transported by boat to the Gump Research Station to be immediately fragmented. Each colony was fragmented into twelve coral nubbins (~5 cm in length), epoxied with Z-Spar to vertically stand on plastic mesh, and distributed to each of mesocosm tanks at 26 °C ± 1 °C for a total of ten nubbins per tank.

After a 24-hour acclimation period, five (half) of the nubbins in each tank were randomly selected and mechanically scarred on the branch tip with 8 mm snub nose pliers. Therefore, each tank contained two treatments: control or scarred with a specific temperature and nutrient regime. (Supplementary Fig. S1). Pliers were sterilized between each scarring to prevent cross-contamination. The resulting scars were ~2 mm deep and removed the tissue layer and portions of the skeletal structure. These scars are representative of corallivory by parrotfishes and pufferfishes, two of the most common scraping corallivores on these reefs. Half of the tanks were then randomly selected and heated to 29 °C (ca. < 1 °C change per hour) to mimic temperatures associated with thermal stress on Mo’orean reefs^47^. Next, each tank was assigned one of three nutrient treatments: pulse of 4 µM of nitrate (NO_3_^−^), pulse of 4 µM of ammonium (NH_4_^+^), or no enrichment controls. For 21 days, each enriched tank was spiked with either nitrate or ammonium twice a day every 12 hr. Flow in all mesocosms was ceased for an hour immediately following the nutrient additions. This resulted in a twice daily hour-long nutrient pulse, followed by 5 hours of steady dilution and 6 hours of ambient concentration.

For microbial analysis, a subset of the nubbins (n = 6 per treatment) were randomly selected while controlling for source tank and colony to minimize samples while ensuring sufficient replication after 21 days of exposure to stressors (Supplementary Fig. S1, Supplementary Table S9). For microbial analysis, a healthy branch tip was clipped off of each unscarred nubbin and the branch tip around the scar was collected for scarred samples. These samples were frozen and shipped to Oregon State University.

### 16S library preparation and sequencing, quality control, and initial data processing

DNA was extracted using the MoBio Powersoil® DNA Isolation Kit according to the manufacturer’s recommendations. Polymerase Chain Reaction (PCR) was performed on the V4 region of the 16S rRNA gene using the primer pair 515F (5′-GTG CCA GCM GCC GCG GTA A-3′) and 806Rb (5′-GGA CTA CHV GGG TWT CTA AT-3′) that targets bacterial and archaeal communities^48,49^. Amplicons were barcoded with barcoding primers with Nextera adapters, pooled in equal volumes for sequencing^50^, and purified with AMPure XP beads. Amplicon pools were paired-end sequenced on the Illumina MiSeq sequencing platform, 2 × 300 bp end version 3 chemistry according to the manufacturer’s specifications at the Oregon State University’s Center for Genome Research and Biocomputing (CGRB) Core Laboratories.

QIIME (v1.9)^51^ was used for quality control and selection of operational taxonomic units (OTUs). Demultiplexed raw reads were trimmed of adapter and primer sequences and pair-end sequences merged. Sequences with a total expected error or total sum of the error probabilities >1 for all bases were discarded. Chimeras were removed and 97%-similarity OTUs were picked using USEARCH 6.1^52^, the 97% GreenGenes 13_8 reference database^53^, and QIIME’s subsampled open-reference OTU-picking protocol^54^. In this protocol, sequences that failed to hit the reference collection are randomly subsampled for de novo clustering. Taxonomy was assigned using UCLUST, and reads were aligned against the GreenGenes database using PyNAST^55^. The GreenGenes reference database is commonly used in microbiome analysis^14^ and has been validated on animal and coral microbiomes in numerous studies^33,56–58^. FastTreeMP^59^ was used to create a bacterial phylogeny with constraints defined by the GreenGenes reference phylogeny.

Both a rarefied and unrarefied OTU table were created for downstream analyses. First, OTUs were filtered out of the starting table if their representative sequences failed to align with PyNAST to the GreenGenes database or if they were annotated as mitochondrial or chloroplast sequences. After this step, the number of reads per sample ranged from 1 to 87262 with a median of 9742 per sample and 3383 unique reads. OTUs with less than 100 counts were then removed from the OTU table resulting in a total of 430 unique reads, and a histogram of reads by samples was plotted (Supplementary Fig. S4). We did not find that any one OTU was associated with one particular sample. Samples with less than 1000 reads were considered undersampled and discarded from the table resulting in an unbalanced experimental design (Supplementary Table S9). Two OTU tables were created from this parent table using the package *phyloseq*^60^ (v1.20.1) in R (v3.4.0). First, the parent table was used as the unrarefied OTU table in downstream dominant taxa and differential OTU abundance analysis. Second, the parent table was rarefied to the minimum sample sequencing depth corresponding to exactly 1070 sequences per sample. This depth was chosen to maximize sample sequencing depth while preserving replication. This rarefied table was then used to calculate a Weighted UniFrac^61,62^ pairwise dissimilarity matrix. Also from this rarefied table, alpha diversity metrics including Faith’s phylogenetic diversity^63^, Chao1 statistic^64^, and Simpson’s diversity index^65^ were calculated in *phyloseq*.

### Statistical analyses investigating alpha and beta diversity

To determine how dominant taxa within the community respond to stressors, the rarefied OTU table was first transformed to proportions to identify the five OTUs with the highest relative abundances. These OTUs were then evaluated for changes with treatment using generalized linear mixed-effects models (GLMM) which allow for inclusion of random effects. Raw OTU counts from the unrarefied table were then included as the response variable in the GLMM with an offset by the log of total sequencing depth to reflect relative abundances. Offset variables are commonly used in count models to adjust for differential exposure times and, for these purposes, to adjust for different sequencing depths. Each OTU count distribution was evaluated for normality with quantile-quantile plots and the Shapiro-Wilk test for normality^66^. A logistic regression with unrarefied count data with an offset was selected over a linear regression with proportion data from the rarefied table since the data were not normally distributed and evidence suggests logistic regressions perform better than arcsine-transformed data in linear regressions^67^. For all models, temperature, nutrients, and scarring were assessed as fixed effects and factorial interaction terms and tank and colony as separate random effects. For the most abundant OTU in the dataset, OTU-Endo, a poisson distribution was used since it resulted in normal residuals using *lme4*^68^ (v1.1.15) and *lmerTest* (v2.0.36) to obtain P-values. For the remaining four OTUs a zero-inflated negative binomial regression was used to account for the excess of zeros in the data using *glmmTMB* (v0.2.2.0). The negative binomial distribution was also evaluated based on normality of residuals. A single zero-inflation parameter was modeled for all observations.

To improve normality of alpha diversity metrics, Chao1 and Faith’s phylogenetic distance were log-transformed, while Simpson’s index was arcsine-transformed. Stressor effects on each alpha diversity metric were assessed with linear mixed effect models (LMM) using *lme4* with temperature, nutrients, and scarring as fixed effects and factorial interaction terms and tank and colony as separate random effects. P-values were approximated with the *lmerTest* (v2.0.36).

For beta diversity analyses, the Weighted Unifrac dissimilarity matrix generated in *phyloseq* (v1.23.1) was used to test for location and dispersion effects acting on the microbial community. First, differences between treatments were assessed with permutational multivariate analysis of variance (PERMANOVA) using the adonis function in the package *vegan* (v2.4.6) both with treatment as the single factor and with pairwise comparisons between treatments^69^. Next, permutation tests for homogeneity in multivariate dispersion (PERMDISP) were performed using the betadisper function in the package *vegan* both with treatment as the single factor and with pairwise comparisons between treatments^70^. For visualizing differences in beta diversity, mean distance-to-centroid values by treatment were extracted from the betadisper results, and mean separation from communities in other treatments or between-group distances were averaged by treatment from the pairwise dissimilarity matrix generated in *phyloseq*. Together, PERMANOVA and PERMDISP provide a comprehensive analysis of deterministic and stochastic changes to the microbiome by evaluating between-group differences and within-group dispersions, respectively. For comparability, the two tests were run with treatment as the single factor, since PERMDISP does not allow for a specified model formula. Pairwise analysis of variance for the PERMANOVA test were conducted using a modified version of *pairwiseAdonis*^71^ (v0.0.1), and for the PERMDISP test with the permutest command in *vegan*. Distance-to-centroid was also regressed against the arcsine-transformed relative abundance of OTU-Endo to determine the correlation between community dispersion and the dominant taxon. Lastly, stressor main effects and interactions were evaluated with PERMANOVA by fitting a linear model to the distance matrices using factorial interaction terms. All diversity analyses were conducted in *R* (v3.4.0).

### Differential OTU abundance analysis

To analyze differences in abundance of bacterial taxa across stressors and stressor interactions, a negative binomial generalized linear model (GLM) was fitted with the R package *DESeq2* (v1.16.1) using the unrarefied OTU table^43^. *DESeq2* does not support random effects and therefore a mixed model was not used. All rare taxa that were present in fewer than 15 samples were excluded from this table to create a pre-filtered, unrarefied OTU table. In the *DESeq2* method, raw counts are modeled with a negative binomial distribution which is commonly used for overdispersed count data^72^. Additionally, “size factors” or normalization factors are calculated with a median-of-ratios method to normalize differences in sequencing depth between samples^43^. The GLM design specified nutrient regime, temperature, scarring, and their interactions as factors with beta priors set to false. Wald post-hoc tests were used to identify factors in the model that significantly affected each taxon compared to the control level by building a results table from a specified treatment contrast. To control the rate of false positives due to multiple comparisons, differentially abundant taxa within each treatment contrast were identified as significant with Benjamini-Hochberg FDR p-values less than 0.05^73^. The pre-filtered, unrarefied OTU table was summarized to Family and Genus level using the tax_glom command in *phyloseq*. The DESeq2 method was then applied to these two tables to demonstrate accordance between significant changes at the OTU level with significant changes at higher taxonomic levels.

## Supplementary information

Supplementary Info

## Data Availability

The datasets generated during and/or analyzed during the current study are available from the corresponding author on reasonable request.

## References

[1] Martínez-Ramos M, Ortiz-Rodríguez IA, Piñero D, Dirzo R, Sarukhán J (2016). Anthropogenic disturbances jeopardize biodiversity conservation within tropical rainforest reserves. Proc. Natl. Acad. Sci..

[2] Galic N, Sullivan L, Grimm V, Forbes VE (2018). When things don’t add up: quantifying impacts of multiple stressors from individual metabolism to ecosystem processing. Ecol. Lett..

[3] Folt CL, Chen CY, Moore MV, Burnaford J (1999). Synergism and antagonism among multiple stressors. Limnol. Oceanogr..

[4] Vinebrooke RD (2004). Impacts of multiple stressors on biodiversity and ecosystem functioning: the role of species co-tolerance. Oikos.

[5] Côté IM, Darling ES, Brown CJ (2016). Interactions among ecosystem stressors and their importance in conservation. Proc. R. Soc. B Biol. Sci..

[6] Brown CJ, Saunders MI, Possingham HP, Richardson AJ (2013). Managing for Interactions between Local and Global Stressors of Ecosystems. PLoS ONE.

[7] Lesser MP, Fiore C, Slattery M, Zaneveld J (2016). Climate change stressors destabilize the microbiome of the Caribbean barrel sponge, Xestospongia muta. J. Exp. Mar. Biol. Ecol..

[8] McDevitt-Irwin JM, Baum JK, Garren M, Vega Thurber RL (2017). Responses of Coral-Associated Bacterial Communities to Local and Global Stressors. Front. Mar. Sci..

[9] Zaneveld JR (2016). Overfishing and nutrient pollution interact with temperature to disrupt coral reefs down to microbial scales. Nat. Commun..

[10] Wang L (2018). Corals and Their Microbiomes Are Differentially Affected by Exposure to Elevated Nutrients and a Natural Thermal Anomaly. Front. Mar. Sci..

[11] Xia Y, Sun J (2017). Hypothesis testing and statistical analysis of microbiome. Genes Dis..

[12] Lewis JD (2015). Inflammation, Antibiotics, and Diet as Environmental Stressors of the Gut Microbiome in Pediatric Crohn’s Disease. Cell Host Microbe.

[13] Lokmer A, Wegner KM (2015). Hemolymph microbiome of Pacific oysters in response to temperature, temperature stress and infection. ISME J..

[14] Knight R (2018). Best practices for analysing microbiomes. Nat. Rev. Microbiol..

[15] Welsh RM (2016). Bacterial predation in a marine host-associated microbiome. ISME J.

[16] Meron D (2011). The impact of reduced pH on the microbial community of the coral Acropora eurystoma. ISME J..

[17] Morrow KM (2015). Natural volcanic CO2 seeps reveal future trajectories for host–microbial associations in corals and sponges. ISME J..

[18] Webster NS (2016). Host-associated coral reef microbes respond to the cumulative pressures of ocean warming and ocean acidification. Sci. Rep..

[19] Gajigan, A. P., Diaz, L. A. & Conaco, C. Resilience of the prokaryotic microbial community of Acropora digitifera to elevated temperature. *MicrobiologyOpen* **6** (2017). 10.1002/mbo3.478 PMC5552946 28425179

[20] Sunagawa S (2009). Bacterial diversity and White Plague Disease-associated community changes in the Caribbean coral *Montastraea faveolata*. ISME J..

[21] Zaneveld JR, McMinds R, Vega Thurber R (2017). Stress and stability: applying the Anna Karenina principle to animal microbiomes. Nat. Microbiol..

[22] Klaus JS, Janse I, Heikoop JM, Sanford RA, Fouke BW (2007). Coral microbial communities, zooxanthellae and mucus along gradients of seawater depth and coastal pollution. Environ. Microbiol..

[23] Rosenberg Eugene, Kushmaro Ariel (2010). Microbial Diseases of Corals: Pathology and Ecology. Coral Reefs: An Ecosystem in Transition.

[24] Ritchie K (2006). Regulation of microbial populations by coral surface mucus and mucus-associated bacteria. Mar. Ecol. Prog. Ser..

[25] Bourne D, Iida Y, Uthicke S, Smith-Keune C (2008). Changes in coral-associated microbial communities during a bleaching event. ISME J..

[26] Thurber RV (2009). Metagenomic analysis of stressed coral holobionts. Environ. Microbiol..

[27] Lee OO (2012). Spatial and species variations in bacterial communities associated with corals from the Red Sea as revealed by pyrosequencing. Appl. Environ. Microbiol..

[28] D. Ainsworth T (2015). The coral core microbiome identifies rare bacterial taxa as ubiquitous endosymbionts. ISME J..

[29] Bayer T (2013). The Microbiome of the Red Sea Coral Stylophora pistillata Is Dominated by Tissue-Associated Endozoicomonas Bacteria. Appl Env. Microbiol.

[30] Shantz AA, Burkepile DE (2014). Context-dependent effects of nutrient loading on the coral–algal mutualism. Ecology.

[31] Goyal A, Dubinkina V, Maslov S (2018). Multiple stable states in microbial communities explained by the stable marriage problem. ISME J..

[32] Garren M, Raymundo L, Guest J, Harvell CD, Azam F (2009). Resilience of Coral-Associated Bacterial Communities Exposed to Fish Farm Effluent. PLoS ONE.

[33] Ziegler M (2016). Coral microbial community dynamics in response to anthropogenic impacts near a major city in the central Red Sea. Mar. Pollut. Bull..

[34] Sweet M, Bythell J (2015). White syndrome in Acropora muricata: nonspecific bacterial infection and ciliate histophagy. Mol. Ecol..

[35] Li, J. *et al*. Bacterial dynamics within the mucus, tissue and skeleton of the coral Porites lutea during different seasons. *Sci. Rep*. **4** (2015). 10.1038/srep07320 PMC4256709 25475855

[36] Koren O, Rosenberg E (2008). Bacteria associated with the bleached and cave coral Oculina patagonica. Microb. Ecol..

[37] Welsh RM (2017). Alien vs. predator: bacterial challenge alters coral microbiomes unless controlled by *Halobacteriovorax* predators. PeerJ.

[38] Pollock FJ, Wada N, Torda G, Willis BL, Bourne DG (2017). White Syndrome-Affected Corals Have a Distinct Microbiome at Disease Lesion Fronts. Appl Env. Microbiol.

[39] Olesen SW, Alm EJ (2016). Dysbiosis is not an answer. Nat. Microbiol..

[40] Duvallet, C., Gibbons, S. M., Gurry, T., Irizarry, R. A. & Alm, E. J. Meta-analysis of gut microbiome studies identifies disease-specific and shared responses. *Nat. Commun*. **8** (2017). 10.1038/s41467-017-01973-8 PMC5716994 29209090

[41] Wipperman MF (2017). Antibiotic treatment for Tuberculosis induces a profound dysbiosis of the microbiome that persists long after therapy is completed. Sci. Rep..

[42] Gurry T (2018). Predictability and persistence of prebiotic dietary supplementation in a healthy human cohort. Sci. Rep..

[43] Love, M. I., Huber, W. & Anders, S. Moderated estimation of fold change and dispersion for RNA-seq data with DESeq2. *Genome Biol*. **15** (2014). 10.1186/s13059-014-0550-8 PMC4302049 25516281

[44] Bosch TCG (2013). Cnidarian-Microbe Interactions and the Origin of Innate Immunity in Metazoans. Annu. Rev. Microbiol..

[45] Bourne DG (2009). Microbial disease and the coral holobiont. Trends Microbiol..

[46] Krediet Cory J, Ritchie Kim B, Paul Valerie J, Max T (2013). Coral-associated micro-organisms and their roles in promoting coral health and thwarting diseases. Proc. R. Soc. B Biol. Sci..

[47] Pratchett MS, McCowan D, Maynard JA, Heron SF (2013). Changes in Bleaching Susceptibility among Corals Subject to Ocean Warming and Recurrent Bleaching in Moorea, French Polynesia. PLoS ONE.

[48] Parada AE, Needham DM, Fuhrman JA (2016). Every base matters: assessing small subunit rRNA primers for marine microbiomes with mock communities, time series and global field samples. Environ. Microbiol..

[49] Apprill A, McNally S, Parsons R, Weber L (2015). Minor revision to V4 region SSU rRNA 806R gene primer greatly increases detection of SAR11 bacterioplankton. Aquat. Microb. Ecol..

[50] Kozich JJ, Westcott SL, Baxter NT, Highlander SK, Schloss PD (2013). Development of a Dual-Index Sequencing Strategy and Curation Pipeline for Analyzing Amplicon Sequence Data on the MiSeq Illumina Sequencing Platform. Appl. Environ. Microbiol..

[51] Caporaso JG (2010). QIIME allows analysis of high-throughput community sequencing data. Nat. Methods.

[52] Edgar RC (2010). Search and clustering orders of magnitude faster than BLAST. Bioinformatics.

[53] McDonald D (2012). An improved Greengenes taxonomy with explicit ranks for ecological and evolutionary analyses of bacteria and archaea. ISME J..

[54] Rideout JR (2014). Subsampled open-reference clustering creates consistent, comprehensive OTU definitions and scales to billions of sequences. PeerJ.

[55] Caporaso JG (2010). PyNAST: a flexible tool for aligning sequences to a template alignment. Bioinformatics.

[56] Morelan, I. A., Gaulke, C. A., Sharpton, T. J., Vega Thurber, R. & Denver, D. R. Microbiome Variation in an Intertidal Sea Anemone Across Latitudes and Symbiotic States. *Front. Mar. Sci*. **6** (2019).

[57] Brown AL, Lipp EK, Osenberg CW (2019). Algae dictate multiple stressor effects on coral microbiomes. Coral Reefs.

[58] Moitinho-Silva L (2017). The sponge microbiome project. GigaScience.

[59] Price MN, Dehal PS, Arkin AP (2010). FastTree 2–approximately maximum-likelihood trees for large alignments. PloS One.

[60] McMurdie PJ, Holmes S (2013). phyloseq: An R Package for Reproducible Interactive Analysis and Graphics of Microbiome Census Data. PLoS ONE.

[61] Lozupone C, Knight R (2005). UniFrac: a New Phylogenetic Method for Comparing Microbial Communities. Appl. Environ. Microbiol..

[62] Lozupone CA, Hamady M, Kelley ST, Knight R (2007). Quantitative and qualitative beta diversity measures lead to different insights into factors that structure microbial communities. Appl. Environ. Microbiol..

[63] Faith DP (1992). Conservation evaluation and phylogenetic diversity. Biol. Conserv..

[64] Chao, A. & Chiu, C.-H. Species Richness: Estimation and Comparison. In *Wiley StatsRef: Statistics Reference Online* (eds Balakrishnan, N. *et al*.) 1–26, 10.1002/9781118445112.stat03432.pub2 (John Wiley & Sons, Ltd, 2016).

[65] Heip, C. H. R., Herman, P. M. J. & Soetaert, K. Indices of diversity and evenness. 27.

[66] Royston JP (1982). An Extension of Shapiro and Wilk’s W Test for Normality to Large Samples. J. R. Stat. Soc. Ser. C Appl. Stat..

[67] Shi PJ, Sand Hu HS, Xiao HJ (2013). Logistic Regression is a better Method of Analysis Than Linear Regression of Arcsine Square Root Transformed Proportional Diapause Data of Pieris melete (Lepidoptera: Pieridae). Fla. Entomol..

[68] Bates D, Mächler M, Bolker B, Walker S (2014). Fitting Linear Mixed-Effects Models Using lme4. J. Stat. Softw..

[69] Anderson MJ (2001). A new method for non-parametric multivariate analysis of variance. Austral Ecol..

[70] Anderson MJ (2006). Distance-Based Tests for Homogeneity of Multivariate Dispersions. Biometrics.

[71] Martinez Arbizu, P. pairwiseAdonis: Pairwise multilevel comparison using adonis. *R Package Version 001* (2017).

[72] Cameron, A. C. & Trivedi, P. K. *Regression Analysis of Count Data*. (Cambridge University Press, 1998).

[73] Benjamini Y, Hochberg Y (1995). Controlling the False Discovery Rate: A Practical and Powerful Approach to Multiple Testing. J. R. Stat. Soc. Ser. B Methodol..

