# Supplementary material for: Multiple stressors interact primarily through antagonism to drive changes in the coral microbiome
Source: Sci Rep. 2019 May 2;9:6834. doi: 10.1038/s41598-019-43274-8 (PMC6497639; doi:10.1038/s41598-019-43274-8)
Supplement: Supplementary file 1 — Supplementary Info [file 41598_2019_43274_MOESM1_ESM.pdf]

**Supplementary Information**

**Title:** Multiple stressors interact primarily through antagonism to drive changes in the coral microbiome

**Authors:** Rebecca L Maher[1]*, Mallory M Rice[2], Ryan McMinds[1], Deron E Burkepile[2,3], and Rebecca Vega Thurber[1].

*Corresponding author

**Email:** beccaluciamaher@gmail.com

[1] Department of Microbiology, Oregon State University, 226 Nash Hall, Corvallis OR 97331, USA

[2] Department of Ecology, Evolution and Marine Biology, University of California Santa Barbara, Santa Barbara CA 93106-9610, USA

[3] Marine Science Institute, University of California Santa Barbara, Santa Barbara CA 93106-9610, USA

## Supplementary Figures

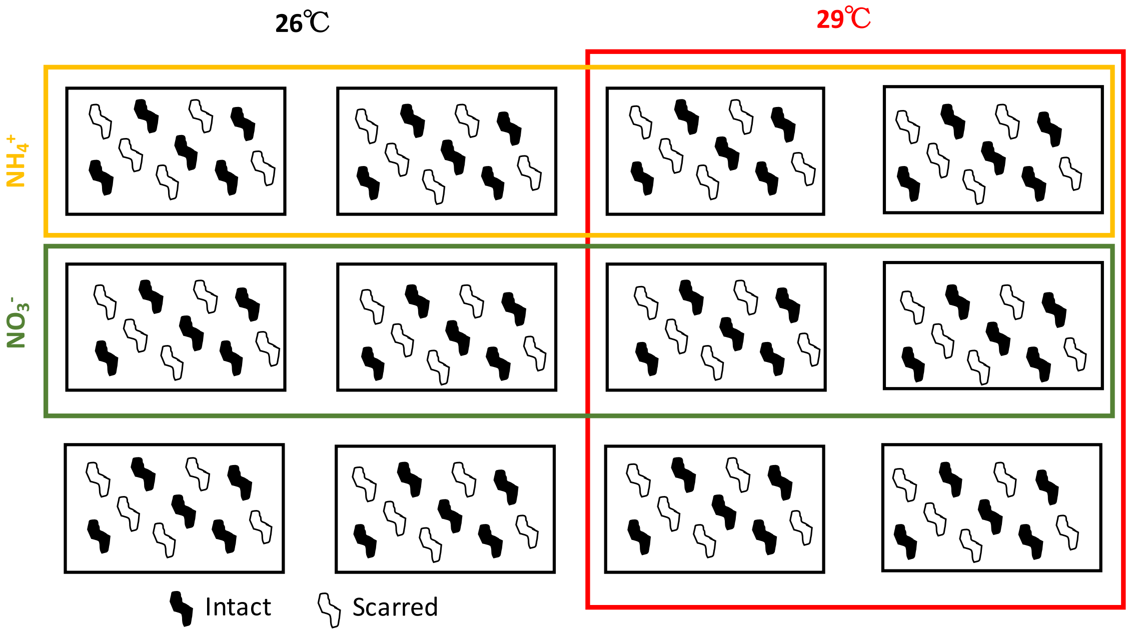


**Supplementary Figure S1.** Tank experimental design. Each tank contained two treatments and each of the twelve treatments was repeated in two tanks, resulting in 10 replicates per treatment. For this study, 6 replicates per treatment were randomly chosen for microbial analyses (3 for each treatment per tank).

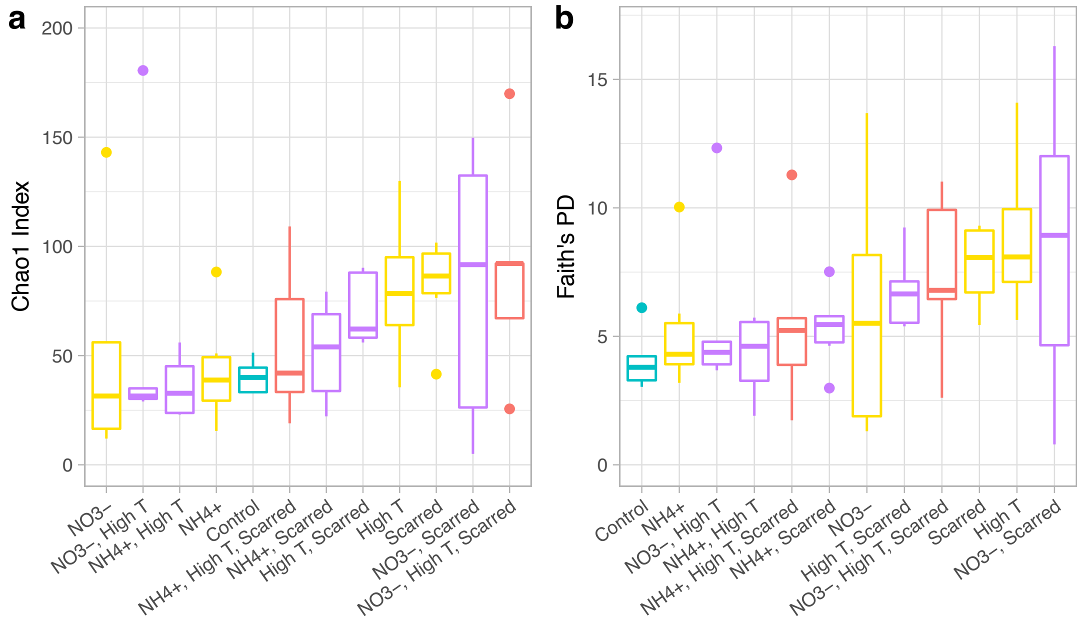


**Supplementary Figure S2.** a) Chao1 index and b) Faith's phylogenetic distance by treatment in order of increasing medians. Colors represent the number of stressors: none=teal, single=yellow, double=purple, triple=red.

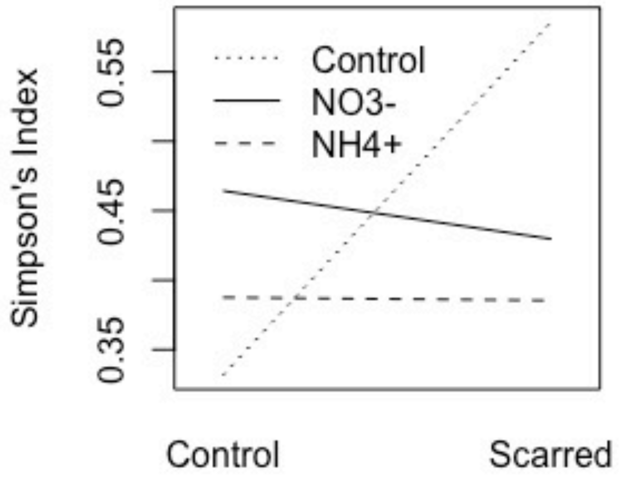


**Supplementary Figure S3.** Interaction plot for Simpson's index by nutrients and scarring treatments. The interaction nutrients:scarring was significant in the linear mixed effects model (Supplementary Table S5).

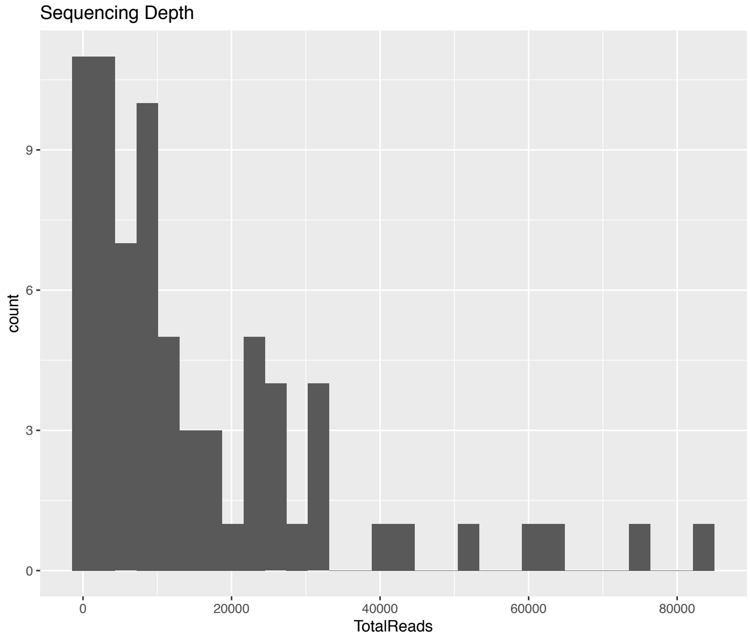


**Supplementary Figure S4.** Histogram of sequencing depth after filtering out OTUs that 1) failed to align with PyNAST to the GreenGenes database, 2) were annotated as mitochondrial or chloroplast sequences, or 3) had less than 100 counts across the entire dataset.

## Supplementary Tables

**Supplementary Table S1. Effects of temperature, nutrients, and scarring on relative abundance of the most abundant OTU, family Endozoicimonaceae**

Generalized linear mixed-effects model of raw counts with an offset for sequencing depth of the most abundant OTU in the dataset. Fixed (temperature, nutrients, and scarring) and random (tank and colony) effects are included in the model. The resulting formula in the R language was: glmer(Endozoicimonaceae ~ offset(log(sequencing_depth)) + temp * nutrient * corallivory + (1 | tank) + (1 | colony), family = “poisson”)

| | OTU, family Endozoicimonaceae | | | |
|---|---|---|---|---|
| **Effect** | **Estimate** | **Std. Error** | **z value** | **P** |
| (Intercept) | -0.07 | 0.07 | -1.04 | 0.30 |
| **High Temp** | **-0.46** | **0.07** | **-6.21** | **<0.001** |
| Nutrient - NH4+ | -0.14 | 0.07 | -1.91 | 0.06 |
| **Nutrient - NO3-** | **-0.15** | **0.07** | **-1.96** | **<0.05** |
| **Scarred** | **-0.52** | **0.01** | **-88.02** | **<0.001** |
| **High Temp:Nutrient - NH4+** | **0.44** | **0.11** | **4.13** | **<0.001** |
| **High Temp: Nutrient - NO3-** | **0.43** | **0.11** | **4.09** | **<0.001** |
| **High Temp: Scarred** | **0.82** | **0.01** | **89.18** | **<0.001** |
| **Nutrient - NH4+:Scarred** | **0.57** | **0.01** | **75.70** | **<0.001** |
| **Nutrient - NO3-:Scarred** | **0.39** | **0.01** | **52.45** | **<0.001** |
| **High Temp:Nutrient - NH4+:Scarred** | **-0.99** | **0.01** | **-68.72** | **<0.001** |
| **High Temp:Nutrient - NO3-:Scarred** | **-0.90** | **0.01** | **-68.72** | **<0.001** |

**Supplementary Table S2. Effects of temperature, nutrients, and scarring on relative abundance of top**

Zero-inflated negative binomial regression for unrarefied count data with an offset for sequencing depth. The offset is included to account for the uneven sequencing depth across the unrarefied table. A single zero-inflation parameter is modeled for all observations. The resulting formula in the R language was: glmmTMB(taxon counts ~ offset(log(sequencing depth)) + temperature*nutrient*temperature + (1|tank) + (1|colony), ziformula = ~1, family = nbinom2)

| | OTU, family Desulfovibrionaceae | | | | OTU, Enterobacteriaceae | | | |
|---|---|---|---|---|---|---|---|---|
| Count model coefficients (negbin with log link): | **Estimate** | **Std. Error** | **z value** | **P** | **Estimate** | **Std. Error** | **z value** | **P** |
| (Intercept) | **-6.84** | **0.86** | **-7.98** | **<0.001** | **-5.48** | **0.5007** | **-10.95** | **<0.001** |
| temphigh | **5.49** | **1.08** | **5.09** | **<0.001** | 1.08 | 0.81 | 1.32 | 0.19 |
| nutrientNH4+ | **3.40** | **1.07** | **3.17** | **<0.01** | 0.99 | 0.75 | 1.32 | 0.19 |
| nutrientNO3- | **5.46** | **1.26** | **4.35** | **<0.001** | -0.11 | 0.92 | -0.13 | 0.90 |
| corallivoryScarred | **4.93** | **1.08** | **4.59** | **<0.001** | **2.38** | **0.7068** | **3.37** | **<0.001** |
| temphigh:nutrientNH4+ | **-6.69** | **1.42** | **-4.71** | **<0.001** | 2.00 | 1.26 | 1.59 | 0.11 |
| temphigh:nutrientNO3- | **-7.24** | **1.53** | **-4.72** | **<0.001** | -0.32 | 1.25 | -0.26 | 0.80 |
| temphigh:corallivoryScarred | **-7.80** | **1.39** | **-5.62** | **<0.001** | -1.56 | 1.08 | -1.45 | 0.15 |
| nutrientNH4+:corallivoryScarred | **-5.84** | **1.54** | **-3.78** | **<0.001** | **-2.08** | **1.03** | **-2.02** | **<0.05** |
| nutrientNO3-:corallivoryScarred | **-6.71** | **1.56** | **-4.31** | **<0.001** | **-3.12** | **1.2734** | **-2.45** | **<0.05** |
| temphigh:nutrientNH4+:corallivoryScarred | **7.51** | **2.02** | **3.72** | **<0.001** | 0.99 | 1.63 | 0.61 | 0.54 |
| temphigh:nutrientNO3-:corallivoryScarred | **9.81** | **1.97** | **4.98** | **<0.001** | 3.21 | 1.70 | 1.89 | 0.06 |
| Zero-inflation parameter (binomial with logit link): | **-1.06** | **0.32** | **-3.26** | **<0.01** | **-1.12** | **0.33** | **-3.44** | **<0.001** |
| | **OTU, family Amoebophilaceae** | | | | **OTU, Moraxellaceae** | | | |
| Count model coefficients (negbin with log link): | **Estimate** | **Std. Error** | **z value** | **P** | **Estimate** | **Std. Error** | **z value** | **P** |
| (Intercept) | **-6.18** | **0.591** | **-10.456** | **<0.001** | **-5.99** | **0.68** | **-8.84** | **<0.001** |
| temphigh | **2.68** | **0.84** | **3.19** | **<0.01** | -0.69 | 1.02 | -0.67 | 0.50 |
| nutrientNH4+ | 0.90 | 0.79 | 1.14 | 0.26 | **1.95** | **0.90** | **2.17** | **<0.05** |
| nutrientNO3- | 0.49 | 0.80 | 0.61 | 0.54 | 1.42 | 0.90 | 1.57 | 0.12 |
| corallivoryScarred | **1.69** | **0.77** | **2.20** | **<0.05** | 1.39 | 0.86 | 1.61 | 0.11 |
| temphigh:nutrientNH4+ | -2.22 | 1.20 | -1.84 | 0.07 | 2.51 | 1.45 | 1.73 | 0.08 |
| temphigh:nutrientNO3- | -0.33 | 1.14 | -0.29 | 0.77 | 0.13 | 1.32 | 0.10 | 0.92 |
| temphigh:corallivoryScarred | -2.20 | 1.15 | -1.92 | 0.06 | 0.46 | 1.27 | 0.36 | 0.72 |
| nutrientNH4+:corallivoryScarred | -1.35 | 1.08 | -1.25 | 0.21 | **-2.95** | **1.23** | **-2.39** | **<0.05** |
| nutrientNO3-:corallivoryScarred | -0.79 | 1.12 | -0.71 | 0.48 | **-3.22** | **1.21** | **-2.67** | **<0.05** |
| temphigh:nutrientNH4+:corallivoryScarred | **3.34** | **1.64** | **2.04** | **<0.05** | -0.12 | 1.86 | -0.06 | 0.95 |
| temphigh:nutrientNO3-:corallivoryScarred | -0.53 | 1.71 | -0.31 | 0.75 | 1.61 | 1.74 | 0.92 | 0.36 |
| Zero-inflation parameter (binomial with logit link): | **-1.79** | **0.44** | **-4.05** | **<0.001** | **-1.15** | **0.35** | **-3.26** | **<0.01** |

**Supplementary Table S3. Effects of temperature, nutrients, and scarring on microbial community alpha diversity metrics**

Linear mixed model of factors affecting coral microbial community richness (Chao1), alpha diversity (Simpson's), and phylogenetic relatedness (Faith's PD) using fixed effects (temperature, nutrients, scarring) and random effects (tank and colony). Chao1 and Faith's PD were log-transformed, while Simpson's Index was arcsine-transformed to improve normality. The resulting formula in the R language was: lmer(alpha diversity metric ~ temperature * nutrients * scarring + (1|tank) + (1|colony)). P-values were approximated with the lmerTest package in R.

| | Chao1 Index | | | | Simpson's Index | | | |
|---|---|---|---|---|---|---|---|---|
| **Effect** | **Estimate** | **Std. Error** | **t value** | **P** | **Estimate** | **Std. Error** | **t value** | **P** |
| (Intercept) | **3.69** | **0.28** | **13.01** | **<0.001** | **0.32** | **0.13** | **2.45** | **<0.05** |
| High Temp | 0.57 | 0.42 | 1.38 | 0.17 | **0.62** | **0.19** | **3.21** | **<0.01** |
| Nutrient - NH4+ | -0.06 | 0.37 | -0.16 | 0.88 | 0.29 | 0.17 | 1.66 | 0.10 |
| Nutrient - NO3- | -0.12 | 0.39 | -0.31 | 0.76 | 0.30 | 0.18 | 1.66 | 0.10 |
| Scarred | 0.68 | 0.37 | 1.81 | 0.08 | **0.67** | **0.17** | **3.82** | **<0.001** |
| High Temp:Nutrient - NH4+ | -0.70 | 0.58 | -1.21 | 0.23 | -0.50 | 0.27 | -1.84 | 0.07 |
| High Temp: Nutrient - NO3- | -0.34 | 0.57 | -0.59 | 0.56 | -0.41 | 0.27 | -1.55 | 0.13 |
| High Temp: Scarred | -0.69 | 0.56 | -1.23 | 0.22 | **-0.84** | **0.26** | **-3.22** | **<0.01** |
| Nutrient - NH4+:Scarred | -0.46 | 0.52 | -0.89 | 0.38 | **-0.72** | **0.24** | **-2.98** | **<0.01** |
| Nutrient - NO3-:Scarred | -0.38 | 0.53 | -0.72 | 0.48 | **-0.63** | **0.25** | **-2.54** | **<0.05** |
| High Temp:Nutrient - NH4+:Scarred | 0.79 | 0.78 | 1.01 | 0.32 | **0.92** | **0.37** | **2.53** | **<0.05** |
| High Temp:Nutrient - NO3-:Scarred | 0.92 | 0.78 | 1.18 | 0.24 | **0.74** | **0.36** | **2.03** | **<0.05** |

| | Faith's Phylogenetic Diversity | | | |
|---|---|---|---|---|
| **Effect** | **Estimate** | **Std. Error** | **t value** | **P** |
| (Intercept) | **1.38** | **0.24** | **5.80** | **<0.001** |
| High Temp | **0.76** | **0.36** | **2.14** | **<0.05** |
| Nutrient - NH4+ | 0.21 | 0.32 | 0.64 | 0.52 |
| Nutrient - NO3- | 0.09 | 0.34 | 0.26 | 0.79 |
| Scarred | **0.65** | **0.32** | **2.04** | **<0.05** |
| High Temp:Nutrient - NH4+ | **-0.99** | **0.49** | **-2.01** | **<0.05** |
| High Temp: Nutrient - NO3- | -0.58 | 0.49 | -1.19 | 0.24 |
| High Temp: Scarred | -0.90 | 0.48 | -1.87 | 0.07 |
| Nutrient - NH4+:Scarred | -0.60 | 0.44 | -1.36 | 0.18 |
| Nutrient - NO3-:Scarred | -0.33 | 0.45 | -0.72 | 0.48 |
| High Temp:Nutrient - NH4+:Scarred | 1.04 | 0.67 | 1.55 | 0.13 |
| High Temp:Nutrient - NO3-:Scarred | 0.81 | 0.67 | 1.21 | 0.23 |

**Supplementary Table S4. Effects of treatment combination on microbial community dissimilarity with pairwise treatment comparisons**

PERMANOVA results for differences between treatments based on dissimilarity measures with the formula: adonis(distance matrix ~ treatment) and p-values for pairwise treatment comparisons.

| **Effect = Treatment** | **DF** | **Sum Sq** | **F** | **$R^2$** | **P** |
|---|---|---|---|---|---|
| Weighted Unifrac | **11** | **1.28** | **1.61** | **0.26** | **<0.05** |

| **Comparison** | **P** |
|---|---|
| **NH4+, High T, Scarred vs Control** | **0.008** |
| NH4+, High T, Scarred vs High T | 0.381 |
| NH4+, High T, Scarred vs High T, Scarred | 0.31 |
| NH4+, High T, Scarred vs NH4+, Scarred | 0.088 |
| NH4+, High T, Scarred vs NO3- | 0.624 |
| NH4+, High T, Scarred vs NH4+ | 0.13 |
| NH4+, High T, Scarred vs NO3-, High T | 0.83 |
| NH4+, High T, Scarred vs NO3-, High T, Scarred | 0.537 |
| NH4+, High T, Scarred vs Scarred | 0.772 |
| NH4+, High T, Scarred vs NO3-, Scarred | 0.34 |
| NH4+, High T, Scarred vs NH4+, High T | 0.868 |
| **Control vs High T** | **0.006** |
| **Control vs High T, Scarred** | **0.008** |
| Control vs NH4+, Scarred | 0.123 |
| Control vs NO3- | 0.171 |
| **Control vs NH4+** | **0.03** |
| **Control vs NO3-, High T** | **0.012** |
| Control vs NO3-, High T, Scarred | 0.059 |
| **Control vs Scarred** | **0.006** |
| Control vs NO3-, Scarred | 0.15 |
| Control vs NH4+, High T | 0.074 |
| High T vs High T, Scarred | 0.058 |
| **High T vs NH4+, Scarred** | **0.02** |
| High T vs NO3- | 0.201 |
| **High T vs NH4+** | **0.022** |
| High T vs NO3-, High T | 0.212 |
| High T vs NO3-, High T, Scarred | 0.166 |
| High T vs Scarred | 0.535 |
| High T vs NO3-, Scarred | 0.136 |
| High T vs NH4+, High T | 0.212 |
| High T, Scarred vs NH4+, Scarred | 0.2 |
| High T, Scarred vs NO3- | 0.647 |

| | |
|---|---|
| High T, Scarred vs NH4+ | 0.229 |
| High T, Scarred vs NO3-, High T | 0.35 |
| High T, Scarred vs NO3-, High T, Scarred | 0.919 |
| High T, Scarred vs Scarred | 0.114 |
| High T, Scarred vs NO3-, Scarred | 0.672 |
| High T, Scarred vs NH4+, High T | 0.285 |
| NH4+, Scarred vs NO3- | 0.375 |
| NH4+, Scarred vs NH4+ | 0.899 |
| NH4+, Scarred vs NO3-, High T | 0.061 |
| NH4+, Scarred vs NO3-, High T, Scarred | 0.327 |
| **NH4+, Scarred vs Scarred** | **0.024** |
| NH4+, Scarred vs NO3-, Scarred | 0.505 |
| NH4+, Scarred vs NH4+, High T | 0.14 |
| NO3- vs NH4+ | 0.771 |
| NO3- vs NO3-, High T | 0.565 |
| NO3- vs NO3-, High T, Scarred | 0.829 |
| NO3- vs Scarred | 0.307 |
| NO3- vs NO3-, Scarred | 0.949 |
| NO3- vs NH4+, High T | 0.427 |
| NH4+ vs NO3-, High T | 0.088 |
| NH4+ vs NO3-, High T, Scarred | 0.486 |
| **NH4+ vs Scarred** | **0.022** |
| NH4+ vs NO3-, Scarred | 0.698 |
| NH4+ vs NH4+, High T | 0.149 |
| NO3-, High T vs NO3-, High T, Scarred | 0.637 |
| NO3-, High T vs Scarred | 0.694 |
| NO3-, High T vs NO3-, Scarred | 0.371 |
| NO3-, High T vs NH4+, High T | 0.403 |
| NO3-, High T, Scarred vs Scarred | 0.335 |
| NO3-, High T, Scarred vs NO3-, Scarred | 0.686 |
| NO3-, High T, Scarred vs NH4+, High T | 0.356 |
| Scarred vs NO3-, Scarred | 0.148 |
| Scarred vs NH4+, High T | 0.416 |
| NO3-, Scarred vs NH4+, High T | 0.351 |

**Supplementary Table S5. Effects of temperature, nutrients, and scarring on microbial community dissimilarity**

PERMANOVA results for differences between groups based Weighted Unifrac dissimilarity measures with the formula: adonis(distance matrix ~ temperature * nutrient * scarring).

| | Weighted Unifrac | | | | |
|---|---|---|---|---|---|
| **Effect** | **DF** | **Sum Sq** | **F** | **$R^2$** | **P** |
| **Temperature** | **1** | **0.25** | **3.39** | **0.05** | **<0.05** |
| Nutrient | 2 | 0.14 | 0.97 | 0.03 | 0.37 |
| Scarring | 1 | 0.04 | 0.60 | 0.01 | 0.61 |
| Temperature:Nutrients | 2 | 0.18 | 1.23 | 0.04 | 0.25 |
| Temperature:Scarring | 1 | 0.18 | 2.46 | 0.04 | 0.05 |
| Nutrients:Scarring | 2 | 0.11 | 0.73 | 0.02 | 0.60 |
| **Temperature:Nutrient:Scarring** | **2** | **0.39** | **2.68** | **0.08** | **<0.05** |

**Supplementary Table S6. Effects of treatment combination on microbial community group dispersion**

PERMDISP results for differences within treatments based on two dissimilarity measures with the formula: betadisper(distance matrix ~ treatment).

| Effect = Treatment | DF | Sum Sq | F | P |
|---|---|---|---|---|
| Weighted Unifrac | **11** | **0.55** | **2.19** | **<0.05** |

| Comparison | P |
|---|---|
| **NH4+, High T, Scarred vs Control** | **0.016** |
| NH4+, High T, Scarred vs High T | 0.944 |
| NH4+, High T, Scarred vs High T, Scarred | 0.153 |
| NH4+, High T, Scarred vs NH4+, Scarred | 0.09 |
| NH4+, High T, Scarred vs NO3- | 0.736 |
| NH4+, High T, Scarred vs NH4+ | 0.115 |
| NH4+, High T, Scarred vs NO3-, High T | 0.577 |
| NH4+, High T, Scarred vs NO3-, High T, Scarred | 0.588 |
| NH4+, High T, Scarred vs Scarred | 0.793 |
| NH4+, High T, Scarred vs NO3-, Scarred | 0.54 |
| NH4+, High T, Scarred vs NH4+, High T | 0.729 |
| **Control vs High T** | **0.005** |
| **Control vs High T, Scarred** | **0.001** |
| **Control vs NH4+, Scarred** | **0.016** |
| **Control vs NO3-** | **0.003** |
| **Control vs NH4+** | **0.012** |
| **Control vs NO3-, High T** | **0.001** |
| **Control vs NO3-, High T, Scarred** | **0.025** |
| **Control vs Scarred** | **0.001** |
| Control vs NO3-, Scarred | 0.105 |
| **Control vs NH4+, High T** | **0.004** |
| High T vs High T, Scarred | 0.057 |
| **High T vs NH4+, Scarred** | **0.037** |
| High T vs NO3- | 0.753 |
| High T vs NH4+ | 0.077 |
| High T vs NO3-, High T | 0.489 |
| High T vs NO3-, High T, Scarred | 0.592 |
| High T vs Scarred | 0.826 |
| High T vs NO3-, Scarred | 0.545 |
| High T vs NH4+, High T | 0.598 |
| High T, Scarred vs NH4+, Scarred | 0.682 |
| High T, Scarred vs NO3- | 0.13 |

| | |
|---|---|
| High T, Scarred vs NH4+ | 0.982 |
| **High T, Scarred vs NO3-, High T** | **0.003** |
| High T, Scarred vs NO3-, High T, Scarred | 0.361 |
| **High T, Scarred vs Scarred** | **0.013** |
| High T, Scarred vs NO3-, Scarred | 0.58 |
| **High T, Scarred vs NH4+, High T** | **0.022** |
| NH4+, Scarred vs NO3- | 0.09 |
| NH4+, Scarred vs NH4+ | 0.733 |
| **NH4+, Scarred vs NO3-, High T** | **0.013** |
| NH4+, Scarred vs NO3-, High T, Scarred | 0.267 |
| **NH4+, Scarred vs Scarred** | **0.01** |
| NH4+, Scarred vs NO3-, Scarred | 0.459 |
| **NH4+, Scarred vs NH4+, High T** | **0.021** |
| NO3- vs NH4+ | 0.146 |
| NO3- vs NO3-, High T | 0.778 |
| NO3- vs NO3-, High T, Scarred | 0.764 |
| NO3- vs Scarred | 0.827 |
| NO3- vs NO3-, Scarred | 0.09 |
| NO3- vs NH4+, High T | 0.417 |
| NH4+ vs NO3-, High T | 0.057 |
| NH4+ vs NO3-, High T, Scarred | 0.34 |
| **NH4+ vs Scarred** | **0.015** |
| NH4+ vs NO3-, Scarred | 0.565 |
| **NH4+ vs NH4+, High T** | **0.031** |
| NO3-, High T vs NO3-, High T, Scarred | 0.888 |
| NO3-, High T vs Scarred | 0.436 |
| NO3-, High T vs NO3-, Scarred | 0.806 |
| NO3-, High T vs NH4+, High T | 0.18 |
| NO3-, High T, Scarred vs Scarred | 0.565 |
| NO3-, High T, Scarred vs NO3-, Scarred | 0.897 |
| NO3-, High T, Scarred vs NH4+, High T | 0.323 |
| Scarred vs NO3-, Scarred | 0.52 |
| Scarred vs NH4+, High T | 0.373 |
| NO3-, Scarred vs NH4+, High T | 0.322 |

**Supplementary Table S7. Effects of stressors on the differences in abundance of bacterial taxa**

Results of a negative binomial generalized linear model (glm) fitted with the R package *DESeq2*. Prior to analysis, the unrarefied OTU table was pre-filtered in phyloseq to excluded all rare taxa that were only present in fewer than 15 samples. The formula in the R language was: ~ temperature * nutrient * scarring. Wald post-hoc tests were performed to identify significant changes and differentially abundant taxa were identified as significant with Benjamini-Hochberg FDR p-values less than 0.05.

| | baseMean | log2 Fold Change | lfcSE | stat | pvalue | padj | Taxonomy | Contrast |
|---|---|---|---|---|---|---|---|---|
| 150582 | 36.83 | 10.61 | 3.05 | 3.47 | 0.001 | 0.009 | k__Bacteria p__Proteobacteria c__Alphaproteobacteria o__ c__Alphaproteobacteria g__ s__ | Control vs High |
| 369965 | 14.43 | 11.70 | 3.83 | 3.05 | 0.002 | 0.024 | k__Bacteria p__Proteobacteria c__Alphaproteobacteria NA c__Alphaproteobacteria NA NA | Control vs High |
| 321533 | 197.96 | 5.87 | 1.87 | 3.14 | 0.002 | 0.024 | k__Bacteria p__Bacteroidetes c__Cytophagia o__Cytophagales f__[Amoebophilaceae] g__SGUS912 s__ | Control vs High |
| 2932342 | 956.20 | 10.65 | 2.45 | 4.35 | 0.000 | 0.000 | k__Bacteria p__Proteobacteria c__Deltaproteobacteria o__Desulfovibrionales f__Desulfovibrionaceae g__ s__ | Control vs High |
| OTU121 | 1.46 | 7.13 | 2.76 | 2.58 | 0.010 | 0.043 | k__Bacteria p__Proteobacteria c__Deltaproteobacteria o__Desulfovibrionales f__Desulfovibrionaceae g__ s__ | Control vs High |
| OTU80 | 4.12 | 7.36 | 2.73 | 2.70 | 0.007 | 0.036 | k__Bacteria p__Proteobacteria c__Deltaproteobacteria o__Desulfovibrionales f__Desulfovibrionaceae g__ s__ | Control vs High |
| OTU123 | 2.07 | 5.66 | 1.92 | 2.95 | 0.003 | 0.024 | k__Bacteria p__Proteobacteria c__Gammaproteobacteria o__Oceanospirillales f__Endozoicimonaceae g__ s__ | Control vs High |
| 1010113 | 122.68 | 18.49 | 3.91 | 4.73 | 0.000 | 0.000 | k__Bacteria p__Proteobacteria c__Gammaproteobacteria o__Enterobacteriales f__Enterobacteriaceae NA NA | Control vs High |
| 165867 | 40.49 | 8.02 | 2.97 | 2.70 | 0.007 | 0.036 | k__Bacteria p__Proteobacteria c__Alphaproteobacteria o__Rhizobiales f__Hyphomicrobiaceae NA NA | Control vs High |
| 428807 | 20.49 | -15.27 | 4.25 | -3.59 | 0.000 | 0.007 | k__Bacteria p__Proteobacteria c__Alphaproteobacteria o__Rhizobiales f__Hyphomicrobiaceae g__ s__ | Control vs High |
| 800197 | 36.52 | 9.91 | 3.58 | 2.77 | 0.006 | 0.034 | k__Bacteria p__Proteobacteria c__Alphaproteobacteria o__Rhodobacterales f__Hyphomonadaceae g__Hyphomonas s__ | Control vs High |
| 933546 | 36.50 | -14.00 | 3.12 | -4.49 | 0.000 | 0.000 | k__Bacteria p__Proteobacteria c__Betaproteobacteria o__Neisseriales f__Neisseriaceae g__ s__ | Control vs High |
| 60398 | 103.95 | 5.41 | 1.95 | 2.78 | 0.005 | 0.034 | k__Bacteria p__Proteobacteria c__Alphaproteobacteria o__Rhodobacterales f__Rhodobacteraceae NA NA | Control vs High |
| 146037 | 5.31 | 12.35 | 4.20 | 2.94 | 0.003 | 0.024 | k__Bacteria p__Proteobacteria c__Alphaproteobacteria o__Rhodobacterales f__Rhodobacteraceae NA NA | Control vs High |
| 163471 | 13.31 | 7.08 | 2.60 | 2.72 | 0.006 | 0.036 | k__Bacteria p__Proteobacteria c__Alphaproteobacteria o__Rhodobacterales f__Rhodobacteraceae NA NA | Control vs High |
| 816411 | 66.85 | 5.60 | 1.86 | 3.01 | 0.003 | 0.024 | k__Bacteria p__Proteobacteria c__Alphaproteobacteria o__Rhodobacterales f__Rhodobacteraceae g__Roseivivax s__ | Control vs High |
| 904675 | 15.56 | 15.13 | 3.71 | 4.08 | 0.000 | 0.001 | k__Bacteria p__Proteobacteria c__Alphaproteobacteria o__Rhodobacterales f__Rhodobacteraceae NA NA | Control vs High |
| 1123147 | 11.28 | 7.70 | 2.54 | 3.02 | 0.002 | 0.024 | k__Bacteria p__Proteobacteria c__Alphaproteobacteria o__Rhodobacterales f__Rhodobacteraceae NA NA | Control vs High |
| 3180137 | 59.37 | 6.70 | 2.26 | 2.97 | 0.003 | 0.024 | k__Bacteria p__Proteobacteria c__Alphaproteobacteria o__Rhodobacterales f__Rhodobacteraceae g__ s__ | Control vs High |
| 431378 | 8.41 | 6.57 | 2.49 | 2.64 | 0.008 | 0.037 | k__Bacteria p__Proteobacteria c__Alphaproteobacteria o__Rhodobacterales f__Rhodobacteraceae NA NA | Control vs High |
| 4327730 | 21.68 | 6.68 | 2.33 | 2.87 | 0.004 | 0.029 | k__Bacteria p__Proteobacteria c__Alphaproteobacteria o__Rhodobacterales f__Rhodobacteraceae NA NA | Control vs High |
| 4420764 | 11.33 | 9.51 | 3.59 | 2.65 | 0.008 | 0.037 | k__Bacteria p__Proteobacteria c__Alphaproteobacteria o__Rhodobacterales f__Rhodobacteraceae g__ s__ | Control vs High |

| | | | | | | | | |
|---|---|---|---|---|---|---|---|---|
| 144589 | 13.01 | 11.48 | 4.32 | 2.66 | 0.008 | 0.037 | k__Bacteria p__Proteobacteria c__Alphaproteobacteria o__Sphingomonadales f__Sphingomonadaceae g__Novosphingobium s__ | Control vs High |
| 833774 | 5.60 | 11.10 | 4.37 | 2.54 | 0.011 | 0.046 | k__Bacteria p__Proteobacteria c__Alphaproteobacteria o__Sphingomonadales f__Sphingomonadaceae g__Novosphingobium s__ | Control vs High |
| 4445466 | 12.88 | -12.68 | 4.18 | -3.03 | 0.002 | 0.024 | k__Bacteria p__Firmicutes c__Bacilli o__Lactobacillales f__Streptococcaceae g__Streptococcus s__ | Control vs High |
| 369965 | 14.43 | 12.01 | 3.44 | 3.49 | 0.000 | 0.013 | k__Bacteria p__Proteobacteria c__Alphaproteobacteria NA c__Alphaproteobacteria NA NA | Control vs NH4 |
| 428807 | 20.49 | -15.80 | 3.83 | -4.13 | 0.000 | 0.001 | k__Bacteria p__Proteobacteria c__Alphaproteobacteria o__Rhizobiales f__Hyphomicrobiaceae g__ s__ | Control vs NH4 |
| 904675 | 15.56 | -11.72 | 3.47 | -3.38 | 0.001 | 0.015 | k__Bacteria p__Proteobacteria c__Alphaproteobacteria o__Rhodobacterales f__Rhodobacteraceae NA NA | Control vs NH4 |
| 3834498 | 6.81 | -17.92 | 3.08 | -5.82 | 0.000 | 0.000 | k__Bacteria p__Proteobacteria c__Alphaproteobacteria o__Rhodobacterales f__Rhodobacteraceae g__Roseivivax s__ | Control vs NH4 |
| 548736 | 7.03 | -16.85 | 3.23 | -5.22 | 0.000 | 0.000 | k__Bacteria p__Proteobacteria c__Alphaproteobacteria o__Rhodobacterales f__Rhodobacteraceae g__Shimia s__ | Control vs NH4 |
| 178785 | 23.84 | -19.19 | 3.57 | -5.38 | 0.000 | 0.000 | k__Bacteria p__Proteobacteria c__Gammaproteobacteria o__Oceanospirillales f__Alcanivoracaceae g__Alcanivorax NA | Control vs NO3 |
| 2932342 | 956.20 | 9.28 | 2.31 | 4.02 | 0.000 | 0.001 | k__Bacteria p__Proteobacteria c__Deltaproteobacteria o__Desulfovibrionales f__Desulfovibrionaceae g__ s__ | Control vs NO3 |
| 321405 | 86.87 | -16.32 | 3.39 | -4.82 | 0.000 | 0.000 | k__Bacteria p__Proteobacteria c__Gammaproteobacteria o__Pseudomonadales f__Moraxellaceae g__Acinetobacter s__lwoffii | Control vs NO3 |
| 3633321 | 359.77 | -20.49 | 2.61 | -7.86 | 0.000 | 0.000 | k__Bacteria p__Proteobacteria c__Gammaproteobacteria o__Pseudomonadales f__Moraxellaceae g__Acinetobacter s__johnsonii | Control vs NO3 |
| 1085703 | 367.78 | -7.36 | 2.47 | -2.98 | 0.003 | 0.023 | k__Bacteria p__Proteobacteria c__Gammaproteobacteria o__Pseudomonadales f__Moraxellaceae g__Acinetobacter s__johnsonii | Control vs NO3 |
| 146037 | 5.31 | 11.80 | 3.95 | 2.99 | 0.003 | 0.023 | k__Bacteria p__Proteobacteria c__Alphaproteobacteria o__Rhodobacterales f__Rhodobacteraceae NA NA | Control vs NO3 |
| 248590 | 7.31 | 10.20 | 3.57 | 2.86 | 0.004 | 0.025 | k__Bacteria p__Proteobacteria c__Alphaproteobacteria o__Rhodobacterales f__Rhodobacteraceae g__Pseudoruegeria s__ | Control vs NO3 |
| 431378 | 8.41 | 6.55 | 2.33 | 2.81 | 0.005 | 0.027 | k__Bacteria p__Proteobacteria c__Alphaproteobacteria o__Rhodobacterales f__Rhodobacteraceae NA NA | Control vs NO3 |
| 1123147 | 11.28 | 7.01 | 2.41 | 2.91 | 0.004 | 0.024 | k__Bacteria p__Proteobacteria c__Alphaproteobacteria o__Rhodobacterales f__Rhodobacteraceae NA NA | Control vs NO3 |
| 4420764 | 11.33 | -12.58 | 3.52 | -3.58 | 0.000 | 0.004 | k__Bacteria p__Proteobacteria c__Alphaproteobacteria o__Rhodobacterales f__Rhodobacteraceae g__ s__ | Control vs NO3 |
| 4421174 | 28.55 | -21.46 | 3.93 | -5.45 | 0.000 | 0.000 | k__Bacteria p__Proteobacteria c__Alphaproteobacteria o__Rhodobacterales f__Rhodobacteraceae NA NA | Control vs NO3 |
| 144589 | 13.01 | 11.96 | 4.07 | 2.94 | 0.003 | 0.024 | k__Bacteria p__Proteobacteria c__Alphaproteobacteria o__Sphingomonadales f__Sphingomonadaceae g__Novosphingobium s__ | Control vs NO3 |
| 833774 | 5.60 | 11.88 | 4.11 | 2.89 | 0.004 | 0.024 | k__Bacteria p__Proteobacteria c__Alphaproteobacteria o__Sphingomonadales f__Sphingomonadaceae g__Novosphingobium s__ | Control vs NO3 |
| 4445466 | 12.88 | -12.80 | 3.93 | -3.26 | 0.001 | 0.011 | k__Bacteria p__Firmicutes c__Bacilli o__Lactobacillales f__Streptococcaceae g__Streptococcus s__ | Control vs NO3 |
| 4393354 | 25.52 | -28.75 | 3.27 | -8.78 | 0.000 | 0.000 | k__Bacteria p__Proteobacteria c__Gammaproteobacteria o__Vibrionales f__Vibrionaceae g__Vibrio s__ | Control vs NO3 |
| 1140185 | 45.17 | -18.54 | 3.47 | -5.34 | 0.000 | 0.000 | k__Bacteria p__Proteobacteria c__Alphaproteobacteria o__Rhodospirillales o__Rhodospirillales NA NA | Control vs NO3 |
| 365755 | 24.16 | -11.89 | 3.29 | -3.61 | 0.000 | 0.004 | k__Bacteria p__Proteobacteria c__Gammaproteobacteria o__Thiohalorhabdales o__Thiohalorhabdales g__ s__ | Control vs NO3 |

| | | | | | | | | |
|---|---|---|---|---|---|---|---|---|
| 150582 | 36.83 | 9.60 | 2.79 | 3.45 | 0.001 | 0.006 | k__Bacteria p__Proteobacteria c__Alphaproteobacteria o__ c__Alphaproteobacteria g__ s__ | Control vs Scarred |
| 369965 | 14.43 | 13.25 | 3.45 | 3.84 | 0.000 | 0.002 | k__Bacteria p__Proteobacteria c__Alphaproteobacteria NA c__Alphaproteobacteria NA NA | Control vs Scarred |
| OTU5 | 33.51 | -17.58 | 3.54 | -4.97 | 0.000 | 0.000 | k__Bacteria p__Proteobacteria c__Alphaproteobacteria NA c__Alphaproteobacteria NA NA | Control vs Scarred |
| 321533 | 197.96 | 4.24 | 1.69 | 2.51 | 0.012 | 0.047 | k__Bacteria p__Bacteroidetes c__Cytophagia o__Cytophagales f__[Amoebophilaceae] g__SGUS912 s__ | Control vs Scarred |
| 2932342 | 956.20 | 8.87 | 2.21 | 4.01 | 0.000 | 0.002 | k__Bacteria p__Proteobacteria c__Deltaproteobacteria o__Desulfovibrionales f__Desulfovibrionaceae g__ s__ | Control vs Scarred |
| OTU121 | 1.46 | 7.11 | 2.51 | 2.83 | 0.005 | 0.025 | k__Bacteria p__Proteobacteria c__Deltaproteobacteria o__Desulfovibrionales f__Desulfovibrionaceae g__ s__ | Control vs Scarred |
| OTU80 | 4.12 | 6.78 | 2.49 | 2.72 | 0.007 | 0.027 | k__Bacteria p__Proteobacteria c__Deltaproteobacteria o__Desulfovibrionales f__Desulfovibrionaceae g__ s__ | Control vs Scarred |
| 1010113 | 122.68 | 14.13 | 3.58 | 3.95 | 0.000 | 0.002 | k__Bacteria p__Proteobacteria c__Gammaproteobacteria o__Enterobacteriales f__Enterobacteriaceae NA NA | Control vs Scarred |
| 165867 | 40.49 | 8.76 | 2.69 | 3.26 | 0.001 | 0.010 | k__Bacteria p__Proteobacteria c__Alphaproteobacteria o__Rhizobiales f__Hyphomicrobiaceae NA NA | Control vs Scarred |
| 149505 | 22.93 | 9.48 | 3.36 | 2.82 | 0.005 | 0.025 | k__Bacteria p__Proteobacteria c__Alphaproteobacteria o__Rhodobacterales f__Hyphomonadaceae g__Oceanicaulis s__ | Control vs Scarred |
| 220012 | 148.66 | 8.61 | 3.08 | 2.80 | 0.005 | 0.025 | k__Bacteria p__Proteobacteria c__Gammaproteobacteria o__Pseudomonadales f__Pseudomonadaceae g__Pseudomonas s__ | Control vs Scarred |
| 60398 | 103.95 | 5.45 | 1.76 | 3.10 | 0.002 | 0.015 | k__Bacteria p__Proteobacteria c__Alphaproteobacteria o__Rhodobacterales f__Rhodobacteraceae NA NA | Control vs Scarred |
| 135260 | 5.93 | 10.03 | 3.64 | 2.75 | 0.006 | 0.026 | k__Bacteria p__Proteobacteria c__Alphaproteobacteria o__Rhodobacterales f__Rhodobacteraceae NA NA | Control vs Scarred |
| 146037 | 5.31 | 14.89 | 3.78 | 3.94 | 0.000 | 0.002 | k__Bacteria p__Proteobacteria c__Alphaproteobacteria o__Rhodobacterales f__Rhodobacteraceae NA NA | Control vs Scarred |
| 163471 | 13.31 | 7.30 | 2.36 | 3.09 | 0.002 | 0.015 | k__Bacteria p__Proteobacteria c__Alphaproteobacteria o__Rhodobacterales f__Rhodobacteraceae NA NA | Control vs Scarred |
| 539299 | 32.63 | 8.95 | 3.18 | 2.82 | 0.005 | 0.025 | k__Bacteria p__Proteobacteria c__Alphaproteobacteria o__Rhodobacterales f__Rhodobacteraceae g__Marivita s__ | Control vs Scarred |
| 816411 | 66.85 | 6.15 | 1.68 | 3.66 | 0.000 | 0.004 | k__Bacteria p__Proteobacteria c__Alphaproteobacteria o__Rhodobacterales f__Rhodobacteraceae g__Roseivivax s__ | Control vs Scarred |
| 904675 | 15.56 | 13.88 | 3.37 | 4.11 | 0.000 | 0.002 | k__Bacteria p__Proteobacteria c__Alphaproteobacteria o__Rhodobacterales f__Rhodobacteraceae NA NA | Control vs Scarred |
| 1123147 | 11.28 | 7.14 | 2.32 | 3.07 | 0.002 | 0.015 | k__Bacteria p__Proteobacteria c__Alphaproteobacteria o__Rhodobacterales f__Rhodobacteraceae NA NA | Control vs Scarred |
| 3180137 | 59.37 | 6.79 | 2.04 | 3.32 | 0.001 | 0.008 | k__Bacteria p__Proteobacteria c__Alphaproteobacteria o__Rhodobacterales f__Rhodobacteraceae g__ s__ | Control vs Scarred |
| 431378 | 8.41 | 5.69 | 2.29 | 2.49 | 0.013 | 0.048 | k__Bacteria p__Proteobacteria c__Alphaproteobacteria o__Rhodobacterales f__Rhodobacteraceae NA NA | Control vs Scarred |
| 4327730 | 21.68 | 6.40 | 2.11 | 3.03 | 0.002 | 0.016 | k__Bacteria p__Proteobacteria c__Alphaproteobacteria o__Rhodobacterales f__Rhodobacteraceae NA NA | Control vs Scarred |
| 4404049 | 8.86 | 11.01 | 3.08 | 3.58 | 0.000 | 0.004 | k__Bacteria p__Proteobacteria c__Alphaproteobacteria o__Rhodobacterales f__Rhodobacteraceae g__Rhodovulum s__ | Control vs Scarred |
| 4420764 | 11.33 | 8.95 | 3.26 | 2.74 | 0.006 | 0.026 | k__Bacteria p__Proteobacteria c__Alphaproteobacteria o__Rhodobacterales f__Rhodobacteraceae g__ s__ | Control vs Scarred |
| 4456340 | 20.14 | 5.49 | 2.11 | 2.60 | 0.009 | 0.038 | k__Bacteria p__Proteobacteria c__Alphaproteobacteria o__Rhodobacterales f__Rhodobacteraceae g__Rhodovulum s__ | Control vs Scarred |
| 144589 | 13.01 | 11.43 | 3.91 | 2.92 | 0.003 | 0.021 | k__Bacteria p__Proteobacteria c__Alphaproteobacteria o__Sphingomonadales f__Sphingomonadaceae g__Novosphingobium s__ | Control vs Scarred |

| | | | | | | | | |
|---|---|---|---|---|---|---|---|---|
| 833774 | 5.60 | 11.00 | 3.96 | 2.78 | 0.005 | 0.026 | k__Bacteria p__Proteobacteria c__Alphaproteobacteria o__Sphingomonadales f__Sphingomonadaceae g__Novosphingobium s__ | Control vs Scarred |
| 365755 | 24.16 | -11.33 | 3.14 | -3.60 | 0.000 | 0.004 | k__Bacteria p__Proteobacteria c__Gammaproteobacteria o__Thiohalorhabdales o__Thiohalorhabdales g__ s__ | Control vs Scarred |
| 2932342 | 956.20 | -14.58 | 3.30 | -4.42 | 0.000 | 0.000 | k__Bacteria p__Proteobacteria c__Deltaproteobacteria o__Desulfovibrionales f__Desulfovibrionaceae g__ s__ | High:scarred |
| OTU80 | 4.12 | -9.60 | 3.64 | -2.64 | 0.008 | 0.045 | k__Bacteria p__Proteobacteria c__Deltaproteobacteria o__Desulfovibrionales f__Desulfovibrionaceae g__ s__ | High:scarred |
| OTU123 | 2.07 | -7.98 | 2.63 | -3.03 | 0.002 | 0.018 | k__Bacteria p__Proteobacteria c__Gammaproteobacteria o__Oceanospirillales f__Endozoicimonaceae g__ s__ | High:scarred |
| 1010113 | 122.68 | -19.41 | 5.25 | -3.70 | 0.000 | 0.003 | k__Bacteria p__Proteobacteria c__Gammaproteobacteria o__Enterobacteriales f__Enterobacteriaceae NA NA | High:scarred |
| 933546 | 36.50 | 16.98 | 4.10 | 4.14 | 0.000 | 0.001 | k__Bacteria p__Proteobacteria c__Betaproteobacteria o__Neisseriales f__Neisseriaceae g__ s__ | High:scarred |
| 5826 | 19.30 | -11.80 | 4.04 | -2.92 | 0.004 | 0.022 | k__Bacteria p__Proteobacteria c__Alphaproteobacteria o__Rhodobacterales f__Rhodobacteraceae g__Rhodovulum s__ | High:scarred |
| 60398 | 103.95 | -7.39 | 2.62 | -2.82 | 0.005 | 0.028 | k__Bacteria p__Proteobacteria c__Alphaproteobacteria o__Rhodobacterales f__Rhodobacteraceae NA NA | High:scarred |
| 146037 | 5.31 | -16.88 | 5.61 | -3.01 | 0.003 | 0.018 | k__Bacteria p__Proteobacteria c__Alphaproteobacteria o__Rhodobacterales f__Rhodobacteraceae NA NA | High:scarred |
| 163471 | 13.31 | -10.05 | 3.47 | -2.89 | 0.004 | 0.023 | k__Bacteria p__Proteobacteria c__Alphaproteobacteria o__Rhodobacterales f__Rhodobacteraceae NA NA | High:scarred |
| 808046 | 39.28 | -22.34 | 4.51 | -4.95 | 0.000 | 0.000 | k__Bacteria p__Proteobacteria c__Alphaproteobacteria o__Rhodobacterales f__Rhodobacteraceae g__ s__ | High:scarred |
| 816411 | 66.85 | -7.83 | 2.50 | -3.13 | 0.002 | 0.016 | k__Bacteria p__Proteobacteria c__Alphaproteobacteria o__Rhodobacterales f__Rhodobacteraceae g__Roseivivax s__ | High:scarred |
| 904675 | 15.56 | -27.14 | 5.02 | -5.41 | 0.000 | 0.000 | k__Bacteria p__Proteobacteria c__Alphaproteobacteria o__Rhodobacterales f__Rhodobacteraceae NA NA | High:scarred |
| 3180137 | 59.37 | -11.84 | 3.07 | -3.86 | 0.000 | 0.002 | k__Bacteria p__Proteobacteria c__Alphaproteobacteria o__Rhodobacterales f__Rhodobacteraceae g__ s__ | High:scarred |
| 4404049 | 8.86 | -29.01 | 4.64 | -6.26 | 0.000 | 0.000 | k__Bacteria p__Proteobacteria c__Alphaproteobacteria o__Rhodobacterales f__Rhodobacteraceae g__Rhodovulum s__ | High:scarred |
| 4420764 | 11.33 | -14.80 | 4.83 | -3.07 | 0.002 | 0.017 | k__Bacteria p__Proteobacteria c__Alphaproteobacteria o__Rhodobacterales f__Rhodobacteraceae g__ s__ | High:scarred |
| 4421174 | 28.55 | -17.37 | 5.62 | -3.09 | 0.002 | 0.017 | k__Bacteria p__Proteobacteria c__Alphaproteobacteria o__Rhodobacterales f__Rhodobacteraceae NA NA | High:scarred |
| 144589 | 13.01 | -20.80 | 5.83 | -3.57 | 0.000 | 0.004 | k__Bacteria p__Proteobacteria c__Alphaproteobacteria o__Sphingomonadales f__Sphingomonadaceae g__Novosphingobium s__ | High:scarred |
| 833774 | 5.60 | -20.13 | 5.90 | -3.41 | 0.001 | 0.007 | k__Bacteria p__Proteobacteria c__Alphaproteobacteria o__Sphingomonadales f__Sphingomonadaceae g__Novosphingobium s__ | High:scarred |
| 4393354 | 25.52 | -19.76 | 4.58 | -4.31 | 0.000 | 0.000 | k__Bacteria p__Proteobacteria c__Gammaproteobacteria o__Vibrionales f__Vibrionaceae g__Vibrio s__ | High:scarred |
| 369965 | 14.43 | -20.23 | 5.31 | -3.81 | 0.000 | 0.002 | k__Bacteria p__Proteobacteria c__Alphaproteobacteria NA c__Alphaproteobacteria NA NA | NH4:high |
| 4478041 | 6.03 | -23.60 | 6.12 | -3.86 | 0.000 | 0.002 | k__Bacteria p__Cyanobacteria c__Oscillatoriophycideae o__Chroococcales f__Cyanobacteriaceae g__Rhopalodia s__gibba | NH4:high |
| 2932342 | 956.20 | -9.11 | 3.40 | -2.68 | 0.007 | 0.043 | k__Bacteria p__Proteobacteria c__Deltaproteobacteria o__Desulfovibrionales f__Desulfovibrionaceae g__ s__ | NH4:high |
| 165867 | 40.49 | -21.82 | 4.25 | -5.14 | 0.000 | 0.000 | k__Bacteria p__Proteobacteria c__Alphaproteobacteria o__Rhizobiales f__Hyphomicrobiaceae NA NA | NH4:high |
| 143362 | 29.69 | 9.99 | 3.17 | 3.15 | 0.002 | 0.015 | k__Bacteria p__Proteobacteria c__Gammaproteobacteria o__Pseudomonadales f__Moraxellaceae g__Acinetobacter s__ | NH4:high |

| | | | | | | | | |
|---|---|---|---|---|---|---|---|---|
| 1085703 | 367.78 | 10.82 | 3.42 | 3.16 | 0.002 | 0.015 | k__Bacteria p__Proteobacteria c__Gammaproteobacteria o__Pseudomonadales f__Moraxellaceae g__Acinetobacter s__johnsonii | NH4:high |
| 3633321 | 359.77 | 9.98 | 3.62 | 2.76 | 0.006 | 0.037 | k__Bacteria p__Proteobacteria c__Gammaproteobacteria o__Pseudomonadales f__Moraxellaceae g__Acinetobacter s__johnsonii | NH4:high |
| 4333705 | 68.73 | 12.13 | 3.21 | 3.78 | 0.000 | 0.002 | k__Bacteria p__Proteobacteria c__Gammaproteobacteria o__Pseudomonadales f__Moraxellaceae g__Acinetobacter NA | NH4:high |
| 933546 | 36.50 | 16.01 | 4.22 | 3.80 | 0.000 | 0.002 | k__Bacteria p__Proteobacteria c__Betaproteobacteria o__Neisseriales f__Neisseriaceae g__ s__ | NH4:high |
| 273239 | 37.01 | -19.63 | 4.95 | -3.97 | 0.000 | 0.002 | k__Bacteria p__Proteobacteria c__Gammaproteobacteria o__Pseudomonadales f__Pseudomonadaceae g__ s__ | NH4:high |
| 548736 | 7.03 | 17.60 | 4.88 | 3.61 | 0.000 | 0.004 | k__Bacteria p__Proteobacteria c__Alphaproteobacteria o__Rhodobacterales f__Rhodobacteraceae g__Shimia s__ | NH4:high |
| 3180137 | 59.37 | -9.34 | 3.20 | -2.92 | 0.003 | 0.026 | k__Bacteria p__Proteobacteria c__Alphaproteobacteria o__Rhodobacterales f__Rhodobacteraceae g__ s__ | NH4:high |
| 4306551 | 56.26 | -21.50 | 4.04 | -5.32 | 0.000 | 0.000 | k__Bacteria p__Proteobacteria c__Alphaproteobacteria o__Rhodobacterales f__Rhodobacteraceae NA NA | NH4:high |
| 4327730 | 21.68 | -9.06 | 3.30 | -2.74 | 0.006 | 0.037 | k__Bacteria p__Proteobacteria c__Alphaproteobacteria o__Rhodobacterales f__Rhodobacteraceae NA NA | NH4:high |
| 4404049 | 8.86 | -19.79 | 4.81 | -4.11 | 0.000 | 0.001 | k__Bacteria p__Proteobacteria c__Alphaproteobacteria o__Rhodobacterales f__Rhodobacteraceae g__Rhodovulum s__ | NH4:high |
| 4421174 | 28.55 | 17.18 | 5.78 | 2.97 | 0.003 | 0.024 | k__Bacteria p__Proteobacteria c__Alphaproteobacteria o__Rhodobacterales f__Rhodobacteraceae NA NA | NH4:high |
| 144589 | 13.01 | -17.79 | 6.01 | -2.96 | 0.003 | 0.024 | k__Bacteria p__Proteobacteria c__Alphaproteobacteria o__Sphingomonadales f__Sphingomonadaceae g__Novosphingobium s__ | NH4:high |
| 833774 | 5.60 | -17.47 | 6.08 | -2.88 | 0.004 | 0.028 | k__Bacteria p__Proteobacteria c__Alphaproteobacteria o__Sphingomonadales f__Sphingomonadaceae g__Novosphingobium s__ | NH4:high |
| 369965 | 14.43 | 30.00 | 7.18 | 4.18 | 0.000 | 0.001 | k__Bacteria p__Proteobacteria c__Alphaproteobacteria NA c__Alphaproteobacteria NA NA | NH4:high:scarred |
| 4478041 | 6.03 | 26.59 | 8.30 | 3.20 | 0.001 | 0.012 | k__Bacteria p__Cyanobacteria c__Oscillatoriophycideae o__Chroococcales f__Cyanobacteriaceae g__Rhopalodia s__gibba | NH4:high:scarred |
| 165867 | 40.49 | 20.99 | 5.69 | 3.69 | 0.000 | 0.003 | k__Bacteria p__Proteobacteria c__Alphaproteobacteria o__Rhizobiales f__Hyphomicrobiaceae NA NA | NH4:high:scarred |
| 933546 | 36.50 | -15.72 | 5.66 | -2.78 | 0.005 | 0.032 | k__Bacteria p__Proteobacteria c__Betaproteobacteria o__Neisseriales f__Neisseriaceae g__ s__ | NH4:high:scarred |
| 200552 | 1298.64 | 13.70 | 4.89 | 2.80 | 0.005 | 0.031 | k__Bacteria p__Proteobacteria c__Gammaproteobacteria o__Pseudomonadales f__Pseudomonadaceae NA NA | NH4:high:scarred |
| 273239 | 37.01 | 26.47 | 6.67 | 3.97 | 0.000 | 0.002 | k__Bacteria p__Proteobacteria c__Gammaproteobacteria o__Pseudomonadales f__Pseudomonadaceae g__ s__ | NH4:high:scarred |
| 146037 | 5.31 | 30.00 | 7.93 | 3.78 | 0.000 | 0.003 | k__Bacteria p__Proteobacteria c__Alphaproteobacteria o__Rhodobacterales f__Rhodobacteraceae NA NA | NH4:high:scarred |
| 808046 | 39.28 | 19.24 | 6.27 | 3.07 | 0.002 | 0.016 | k__Bacteria p__Proteobacteria c__Alphaproteobacteria o__Rhodobacterales f__Rhodobacteraceae g__ s__ | NH4:high:scarred |
| 3180137 | 59.37 | 15.12 | 4.36 | 3.47 | 0.001 | 0.005 | k__Bacteria p__Proteobacteria c__Alphaproteobacteria o__Rhodobacterales f__Rhodobacteraceae g__ s__ | NH4:high:scarred |
| 4306551 | 56.26 | 23.72 | 5.43 | 4.37 | 0.000 | 0.001 | k__Bacteria p__Proteobacteria c__Alphaproteobacteria o__Rhodobacterales f__Rhodobacteraceae NA NA | NH4:high:scarred |
| 4327730 | 21.68 | 14.29 | 4.46 | 3.20 | 0.001 | 0.012 | k__Bacteria p__Proteobacteria c__Alphaproteobacteria o__Rhodobacterales f__Rhodobacteraceae NA NA | NH4:high:scarred |
| 4421174 | 28.55 | 22.33 | 7.88 | 2.84 | 0.005 | 0.030 | k__Bacteria p__Proteobacteria c__Alphaproteobacteria o__Rhodobacterales f__Rhodobacteraceae NA NA | NH4:high:scarred |

| | | | | | | | | |
|---|---|---|---|---|---|---|---|---|
| 144589 | 13.01 | 30.00 | 8.13 | 3.69 | 0.000 | 0.003 | k__Bacteria p__Proteobacteria c__Alphaproteobacteria o__Sphingomonadales f__Sphingomonadaceae g__Novosphingobium s__ | NH4:high:scarred |
| 833774 | 5.60 | 30.00 | 8.23 | 3.64 | 0.000 | 0.003 | k__Bacteria p__Proteobacteria c__Alphaproteobacteria o__Sphingomonadales f__Sphingomonadaceae g__Novosphingobium s__ | NH4:high:scarred |
| 1040220 | 6654.92 | 20.69 | 6.72 | 3.08 | 0.002 | 0.016 | k__Bacteria p__Firmicutes c__Bacilli o__Bacillales f__Staphylococcaceae g__Staphylococcus s__ | NH4:high:scarred |
| 4345285 | 608.03 | 21.86 | 7.32 | 2.99 | 0.003 | 0.020 | k__Bacteria p__Firmicutes c__Bacilli o__Bacillales f__Staphylococcaceae g__Staphylococcus s__ | NH4:high:scarred |
| 4445466 | 12.88 | 30.00 | 7.82 | 3.84 | 0.000 | 0.003 | k__Bacteria p__Firmicutes c__Bacilli o__Lactobacillales f__Streptococcaceae g__Streptococcus s__ | NH4:high:scarred |
| 4393354 | 25.52 | 30.00 | 6.47 | 4.63 | 0.000 | 0.000 | k__Bacteria p__Proteobacteria c__Gammaproteobacteria o__Vibrionales f__Vibrionaceae g__Vibrio s__ | NH4:high:scarred |
| 369965 | 14.43 | -24.29 | 4.76 | -5.10 | 0.000 | 0.000 | k__Bacteria p__Proteobacteria c__Alphaproteobacteria NA c__Alphaproteobacteria NA NA | NH4:scarred |
| 2932342 | 956.20 | -10.81 | 3.05 | -3.54 | 0.000 | 0.008 | k__Bacteria p__Proteobacteria c__Deltaproteobacteria o__Desulfovibrionales f__Desulfovibrionaceae g__ s__ | NH4:scarred |
| 800197 | 36.52 | -12.79 | 4.49 | -2.85 | 0.004 | 0.047 | k__Bacteria p__Proteobacteria c__Alphaproteobacteria o__Rhodobacterales f__Hyphomonadaceae g__Hyphomonas s__ | NH4:scarred |
| 146037 | 5.31 | -19.77 | 5.28 | -3.74 | 0.000 | 0.005 | k__Bacteria p__Proteobacteria c__Alphaproteobacteria o__Rhodobacterales f__Rhodobacteraceae NA NA | NH4:scarred |
| 3180137 | 59.37 | -9.28 | 2.83 | -3.28 | 0.001 | 0.018 | k__Bacteria p__Proteobacteria c__Alphaproteobacteria o__Rhodobacterales f__Rhodobacteraceae g__ s__ | NH4:scarred |
| 4327730 | 21.68 | -9.12 | 2.94 | -3.11 | 0.002 | 0.028 | k__Bacteria p__Proteobacteria c__Alphaproteobacteria o__Rhodobacterales f__Rhodobacteraceae NA NA | NH4:scarred |
| 4421174 | 28.55 | -15.92 | 5.20 | -3.06 | 0.002 | 0.028 | k__Bacteria p__Proteobacteria c__Alphaproteobacteria o__Rhodobacterales f__Rhodobacteraceae NA NA | NH4:scarred |
| 548736 | 7.03 | 16.53 | 4.40 | 3.76 | 0.000 | 0.005 | k__Bacteria p__Proteobacteria c__Alphaproteobacteria o__Rhodobacterales f__Rhodobacteraceae g__Shimia s__ | NH4:scarred |
| 4445466 | 12.88 | -28.42 | 5.13 | -5.54 | 0.000 | 0.000 | k__Bacteria p__Firmicutes c__Bacilli o__Lactobacillales f__Streptococcaceae g__Streptococcus s__ | NH4:scarred |
| 365755 | 24.16 | 12.13 | 4.27 | 2.84 | 0.004 | 0.047 | k__Bacteria p__Proteobacteria c__Gammaproteobacteria o__Thiohalorhabdales o__Thiohalorhabdales g__ s__ | NH4:scarred |
| 178785 | 23.84 | 17.61 | 5.11 | 3.45 | 0.001 | 0.007 | k__Bacteria p__Proteobacteria c__Gammaproteobacteria o__Oceanospirillales f__Alcanivoracaceae g__Alcanivorax NA | NO3:high |
| 2932342 | 956.20 | -12.60 | 3.36 | -3.75 | 0.000 | 0.003 | k__Bacteria p__Proteobacteria c__Deltaproteobacteria o__Desulfovibrionales f__Desulfovibrionaceae g__ s__ | NO3:high |
| 428807 | 20.49 | 22.30 | 5.83 | 3.82 | 0.000 | 0.003 | k__Bacteria p__Proteobacteria c__Alphaproteobacteria o__Rhizobiales f__Hyphomicrobiaceae g__ s__ | NO3:high |
| 321405 | 86.87 | 18.03 | 4.83 | 3.73 | 0.000 | 0.003 | k__Bacteria p__Proteobacteria c__Gammaproteobacteria o__Pseudomonadales f__Moraxellaceae g__Acinetobacter s__lwoffii | NO3:high |
| 3633321 | 359.77 | 22.77 | 3.69 | 6.17 | 0.000 | 0.000 | k__Bacteria p__Proteobacteria c__Gammaproteobacteria o__Pseudomonadales f__Moraxellaceae g__Acinetobacter s__johnsonii | NO3:high |
| 933546 | 36.50 | 18.73 | 4.16 | 4.50 | 0.000 | 0.000 | k__Bacteria p__Proteobacteria c__Betaproteobacteria o__Neisseriales f__Neisseriaceae g__ s__ | NO3:high |
| 4421174 | 28.55 | 28.24 | 5.75 | 4.91 | 0.000 | 0.000 | k__Bacteria p__Proteobacteria c__Alphaproteobacteria o__Rhodobacterales f__Rhodobacteraceae NA NA | NO3:high |
| 144589 | 13.01 | -16.20 | 5.90 | -2.74 | 0.006 | 0.050 | k__Bacteria p__Proteobacteria c__Alphaproteobacteria o__Sphingomonadales f__Sphingomonadaceae g__Novosphingobium s__ | NO3:high |
| 833774 | 5.60 | -16.64 | 5.99 | -2.78 | 0.005 | 0.049 | k__Bacteria p__Proteobacteria c__Alphaproteobacteria o__Sphingomonadales f__Sphingomonadaceae g__Novosphingobium s__ | NO3:high |
| 4445466 | 12.88 | 30.00 | 5.73 | 5.23 | 0.000 | 0.000 | k__Bacteria p__Firmicutes c__Bacilli o__Lactobacillales f__Streptococcaceae g__Streptococcus s__ | NO3:high |

| | | | | | | | | |
|---|---|---|---|---|---|---|---|---|
| 1140185 | 45.17 | 14.51 | 4.98 | 2.91 | 0.004 | 0.035 | k__Bacteria p__Proteobacteria c__Alphaproteobacteria o__Rhodospirillales o__Rhodospirillales NA NA | NO3:high |
| 365755 | 24.16 | -15.96 | 4.78 | -3.34 | 0.001 | 0.009 | k__Bacteria p__Proteobacteria c__Gammaproteobacteria o__Thiohalorhabdales o__Thiohalorhabdales g__ s__ | NO3:high |
| 2932342 | 956.20 | 18.68 | 4.59 | 4.07 | 0.000 | 0.002 | k__Bacteria p__Proteobacteria c__Deltaproteobacteria o__Desulfovibrionales f__Desulfovibrionaceae g__ s__ | NO3:high:scarred |
| 1010113 | 122.68 | 30.00 | 7.32 | 4.10 | 0.000 | 0.002 | k__Bacteria p__Proteobacteria c__Gammaproteobacteria o__Enterobacteriales f__Enterobacteriaceae NA NA | NO3:high:scarred |
| 428807 | 20.49 | -22.70 | 7.83 | -2.90 | 0.004 | 0.035 | k__Bacteria p__Proteobacteria c__Alphaproteobacteria o__Rhizobiales f__Hyphomicrobiaceae g__ s__ | NO3:high:scarred |
| 933546 | 36.50 | -17.77 | 5.61 | -3.17 | 0.002 | 0.016 | k__Bacteria p__Proteobacteria c__Betaproteobacteria o__Neisseriales f__Neisseriaceae g__ s__ | NO3:high:scarred |
| 337713 | 13.70 | 29.92 | 8.02 | 3.73 | 0.000 | 0.004 | k__Bacteria p__Planctomycetes c__Planctomycetia o__Pirellulales f__Pirellulaceae g__ s__ | NO3:high:scarred |
| 200552 | 1298.64 | 13.97 | 4.91 | 2.84 | 0.004 | 0.039 | k__Bacteria p__Proteobacteria c__Gammaproteobacteria o__Pseudomonadales f__Pseudomonadaceae NA NA | NO3:high:scarred |
| 808046 | 39.28 | 21.75 | 6.22 | 3.50 | 0.000 | 0.008 | k__Bacteria p__Proteobacteria c__Alphaproteobacteria o__Rhodobacterales f__Rhodobacteraceae g__ s__ | NO3:high:scarred |
| 904675 | 15.56 | 30.00 | 6.98 | 4.30 | 0.000 | 0.002 | k__Bacteria p__Proteobacteria c__Alphaproteobacteria o__Rhodobacterales f__Rhodobacteraceae NA NA | NO3:high:scarred |
| 4404049 | 8.86 | 24.17 | 6.39 | 3.78 | 0.000 | 0.004 | k__Bacteria p__Proteobacteria c__Alphaproteobacteria o__Rhodobacterales f__Rhodobacteraceae g__Rhodovulum s__ | NO3:high:scarred |
| 144589 | 13.01 | 26.73 | 8.05 | 3.32 | 0.001 | 0.010 | k__Bacteria p__Proteobacteria c__Alphaproteobacteria o__Sphingomonadales f__Sphingomonadaceae g__Novosphingobium s__ | NO3:high:scarred |
| 833774 | 5.60 | 27.10 | 8.16 | 3.32 | 0.001 | 0.010 | k__Bacteria p__Proteobacteria c__Alphaproteobacteria o__Sphingomonadales f__Sphingomonadaceae g__Novosphingobium s__ | NO3:high:scarred |
| 4393354 | 25.52 | 17.81 | 6.46 | 2.76 | 0.006 | 0.046 | k__Bacteria p__Proteobacteria c__Gammaproteobacteria o__Vibrionales f__Vibrionaceae g__Vibrio s__ | NO3:high:scarred |
| 593848 | 3284.32 | 17.19 | 5.06 | 3.39 | 0.001 | 0.010 | k__Bacteria p__Proteobacteria c__Gammaproteobacteria o__Xanthomonadales f__Xanthomonadaceae g__Stenotrophomonas s__ | NO3:high:scarred |
| OTU5 | 33.51 | 22.02 | 4.94 | 4.46 | 0.000 | 0.000 | k__Bacteria p__Proteobacteria c__Alphaproteobacteria NA c__Alphaproteobacteria NA NA | NO3:scarred |
| 2932342 | 956.20 | -11.56 | 3.12 | -3.70 | 0.000 | 0.002 | k__Bacteria p__Proteobacteria c__Deltaproteobacteria o__Desulfovibrionales f__Desulfovibrionaceae g__ s__ | NO3:scarred |
| 1010113 | 122.68 | -23.35 | 5.08 | -4.59 | 0.000 | 0.000 | k__Bacteria p__Proteobacteria c__Gammaproteobacteria o__Enterobacteriales f__Enterobacteriaceae NA NA | NO3:scarred |
| 3633321 | 359.77 | 12.60 | 3.48 | 3.63 | 0.000 | 0.003 | k__Bacteria p__Proteobacteria c__Gammaproteobacteria o__Pseudomonadales f__Moraxellaceae g__Acinetobacter s__johnsonii | NO3:scarred |
| 337713 | 13.70 | -23.61 | 5.46 | -4.32 | 0.000 | 0.000 | k__Bacteria p__Planctomycetes c__Planctomycetia o__Pirellulales f__Pirellulaceae g__ s__ | NO3:scarred |
| 200552 | 1298.64 | -13.12 | 3.38 | -3.89 | 0.000 | 0.001 | k__Bacteria p__Proteobacteria c__Gammaproteobacteria o__Pseudomonadales f__Pseudomonadaceae NA NA | NO3:scarred |
| 220012 | 148.66 | -12.72 | 4.36 | -2.92 | 0.003 | 0.024 | k__Bacteria p__Proteobacteria c__Gammaproteobacteria o__Pseudomonadales f__Pseudomonadaceae g__Pseudomonas s__ | NO3:scarred |
| 256116 | 494.08 | -11.43 | 3.38 | -3.38 | 0.001 | 0.006 | k__Bacteria p__Proteobacteria c__Gammaproteobacteria o__Pseudomonadales f__Pseudomonadaceae g__ s__ | NO3:scarred |
| 146037 | 5.31 | -15.96 | 5.30 | -3.01 | 0.003 | 0.019 | k__Bacteria p__Proteobacteria c__Alphaproteobacteria o__Rhodobacterales f__Rhodobacteraceae NA NA | NO3:scarred |
| 4404049 | 8.86 | -11.52 | 4.27 | -2.70 | 0.007 | 0.045 | k__Bacteria p__Proteobacteria c__Alphaproteobacteria o__Rhodobacterales f__Rhodobacteraceae g__Rhodovulum s__ | NO3:scarred |
| 4421174 | 28.55 | 22.62 | 5.26 | 4.30 | 0.000 | 0.000 | k__Bacteria p__Proteobacteria c__Alphaproteobacteria o__Rhodobacterales f__Rhodobacteraceae NA NA | NO3:scarred |

| | | | | | | | | |
|---|---|---|---|---|---|---|---|---|
| 144589 | 13.01 | -14.67 | 5.47 | -2.68 | 0.007 | 0.045 | k__Bacteria p__Proteobacteria c__Alphaproteobacteria o__Sphingomonadales f__Sphingomonadaceae g__Novosphingobium s__ | NO3:scarred |
| 4393354 | 25.52 | 29.63 | 4.35 | 6.81 | 0.000 | 0.000 | k__Bacteria p__Proteobacteria c__Gammaproteobacteria o__Vibrionales f__Vibrionaceae g__Vibrio s__ | NO3:scarred |
| 593848 | 3284.32 | -10.54 | 3.46 | -3.05 | 0.002 | 0.019 | k__Bacteria p__Proteobacteria c__Gammaproteobacteria o__Xanthomonadales f__Xanthomonadaceae g__Stenotrophomonas s__ | NO3:scarred |
| 1140185 | 45.17 | 18.35 | 4.62 | 3.97 | 0.000 | 0.001 | k__Bacteria p__Proteobacteria c__Alphaproteobacteria o__Rhodospirillales o__Rhodospirillales NA NA | NO3:scarred |
| 365755 | 24.16 | 30.00 | 4.45 | 6.74 | 0.000 | 0.000 | k__Bacteria p__Proteobacteria c__Gammaproteobacteria o__Thiohalorhabdales o__Thiohalorhabdales g__ s__ | NO3:scarred |

**Supplementary Table S8. Comparison of differential abundance analysis results from OTU tables summarized at the Family, Genus, and OTU levels.**
Family and Genus tables were obtained using the tax_glom function in *phyloseq* on the unrarefied input data to DESeq2 analysis presented in Supplementary Table S8. All results are presented as log-fold changes that were significant at an adjusted p-value of <0.05. Significant families are labeled at the family level. Significant genera are listed under their corresponding family and labeled by Genus. Significant OTUs are listed under their corresponding genus and are labeled by Species.

| Taxonomy | | | Comparison | | | | | | | | | | |
|---|---|---|---|---|---|---|---|---|---|---|---|---|---|
| Family | Genus | Species | Control vs. High T | Control vs. Scar | Control vs. NO3 | Control vs. NH4 | High T: Scar | NO3: Scar | NH4: Scar | NO3: High T | NH4: High T | NO3:High T :Scar | NH4:High T :Scar |
| Class: Alphaproteobacteria | | | - | 30.00 | -24.25 | 22.61 | - | 24.00 | 30.00 | 30.00 | -30.00 | 30.00 | |
| | g__ | | -20.55 | - | - | -15.52 | 23.49 | -30.00 | 30.00 | 30.00 | - | NA | NA |
| | | s__ | 11.70 | 13.25 | - | 12.01 | - | - | -24.29 | - | -20.23 | - | 30.00 |
| | | s__ | - | -17.58 | - | - | - | 22.02 | - | - | - | - | - |
| | | s__ | 10.61 | 9.60 | - | - | - | - | - | - | - | - | - |
| c__Alphaproteobacteria; o__BD7-3 | | | -30.00 | 21.60 | -30.00 | - | 21.56 | -30.00 | -30.00 | - | -30.00 | - | |
| | g__ | | - | - | - | -15.65 | 30.00 | -30.00 | -28.93 | 26.82 | - | NA | NA |
| c__Gammaproteobacteria; o__Thiohalorhabdales | | | -16.09 | - | -30.00 | 30.00 | - | -30.00 | 30.00 | -30.00 | - | - | |
| | g__ | | - | -16.26 | -30.00 | - | 30.00 | - | 27.33 | -30.00 | - | NA | NA |
| | | s__ | - | -11.33 | -11.89 | - | - | 30.00 | 12.13 | -15.96 | - | - | - |
| f__[Amoebophilaceae] | | | 30.00 | 30.00 | 30.00 | 30.00 | -30.00 | 30.00 | 24.78 | - | - | -30.00 | |
| | g__SGUS912 | | - | 30.00 | 30.00 | 30.00 | -30.00 | 30.00 | -30.00 | - | -30.00 | NA | NA |
| | | s__ | 5.87 | 4.24 | - | - | - | - | - | - | - | - | - |
| f__Alcanivoracaceae | | | 30.00 | 30.00 | -30.00 | 30.00 | -30.00 | -30.00 | -30.00 | -30.00 | - | -30.00 | |
| | g__Alcanivorax | | - | - | -30.00 | - | - | -30.00 | -30.00 | - | - | NA | NA |
| | | NA | - | - | -19.19 | - | - | - | - | 17.61 | - | - | - |
| f__Cohaesibacteraceae | | | -30.00 | -30.00 | -30.00 | -22.68 | - | -21.08 | -22.08 | - | - | 30.00 | |
| | g__Cohaesibacter | | - | - | - | - | - | -30.00 | - | - | - | NA | NA |
| f__Cyanobacteriaceae | | | 17.39 | - | -25.91 | -30.00 | - | - | - | - | - | 30.00 | |
| | g__Rhopalodia | | - | - | -30.00 | -30.00 | -27.12 | - | - | 24.90 | - | NA | NA |
| | | s__gibba | - | - | - | - | - | - | - | - | -23.60 | - | 26.59 |

| | | | | | | | | | | | | | |
|---|---|---|---|---|---|---|---|---|---|---|---|---|---|
| f__Desulfovibrionaceae | | | 30.00 | 30.00 | 30.00 | 30.00 | -30.00 | -30.00 | - | 25.29 | - | - | |
| | g__ | | - | - | 30.00 | 30.00 | - | -30.00 | -30.00 | - | -30.00 | NA | NA |
| | | s__ | 10.65 | 8.87 | 9.28 | - | -14.58 | -11.56 | -10.81 | -12.60 | -9.11 | 18.68 | - |
| | | s__ | 7.13 | 7.11 | - | - | - | - | - | - | - | - | - |
| | | s__ | 7.36 | 6.78 | - | - | -9.60 | - | - | - | - | - | - |
| f__Endozoicimonaceae | | | 30.00 | 30.00 | 30.00 | 30.00 | -30.00 | 30.00 | 30.00 | 25.30 | - | -30.00 | |
| | g__ | | - | 30.00 | 30.00 | 30.00 | -30.00 | 30.00 | - | - | -30.00 | NA | NA |
| | | s__ | 5.66 | - | - | - | -7.98 | - | - | - | - | - | - |
| f__Enterobacteriaceae | | | 30.00 | 30.00 | 30.00 | 30.00 | -30.00 | 30.00 | 26.07 | - | - | -30.00 | |
| | NA | | - | 30.00 | 30.00 | 30.00 | -30.00 | 30.00 | -30.00 | - | -28.47 | NA | NA |
| | | NA | 18.49 | 14.13 | - | - | -19.41 | -23.35 | - | - | - | 30.00 | - |
| f__Enterobacteriaceae | g__Klebsiella | | - | 30.00 | 30.00 | 30.00 | -30.00 | 30.00 | -30.00 | - | -24.38 | NA | NA |
| f__Flavobacteriaceae | | | 20.27 | - | - | -21.56 | 30.00 | - | - | 30.00 | - | 30.00 | |
| | g__Ascidianibacter | | - | - | - | -24.46 | - | -26.07 | 30.00 | 30.00 | -21.61 | NA | NA |
| | NA | | - | - | - | -30.00 | 30.00 | -30.00 | - | -30.00 | - | NA | NA |
| f__Hyphomicrobiaceae | | | 30.00 | 24.64 | -30.00 | 30.00 | -30.00 | - | 30.00 | 30.00 | -30.00 | 30.00 | |
| | NA | | 27.37 | - | - | 16.34 | -24.81 | -30.00 | 30.00 | - | -30.00 | NA | NA |
| | | NA | 8.02 | 8.76 | - | - | - | - | - | - | -21.82 | - | 20.99 |
| | g__ | | -30.00 | - | - | -30.00 | 21.94 | -30.00 | - | - | - | NA | NA |
| | | s__ | -15.27 | - | - | -15.80 | - | - | - | 22.30 | - | -22.70 | - |
| f__Hyphomonadaceae | | | - | -30.00 | -30.00 | 30.00 | 30.00 | 27.37 | -30.00 | 30.00 | 25.64 | 30.00 | |
| | g__Oceanicaulis | | - | 21.76 | -30.00 | -30.00 | 30.00 | - | - | - | - | NA | NA |
| | | s__ | - | 9.48 | - | - | - | - | - | - | - | - | - |
| | g__Hyphomonas | | -30.00 | - | - | 23.67 | - | -27.36 | -30.00 | - | 25.59 | NA | NA |
| | | s__ | 9.91 | - | - | - | - | - | -12.79 | - | - | - | - |
| f__Moraxellaceae | | | 20.94 | 30.00 | 30.00 | 30.00 | -30.00 | 30.00 | 25.91 | 30.00 | - | -30.00 | |
| | g__Acinetobacter | | - | 30.00 | 30.00 | 30.00 | -30.00 | 30.00 | -29.38 | - | - | NA | NA |

| | | | | | | | | | | | | | |
|---|---|---|---|---|---|---|---|---|---|---|---|---|---|
| | | NA | - | - | - | - | - | - | - | - | 12.13 | - | - |
| | | s__ | - | - | - | - | - | - | - | - | 9.99 | - | - |
| | | s__john sonii | - | - | -7.36 | - | - | - | - | - | 10.82 | - | - |
| | | s__john sonii | - | - | -20.49 | - | - | 12.60 | - | 22.77 | 9.98 | - | - |
| | | s__lwoff ii | - | - | -16.32 | - | - | - | - | 18.03 | - | - | - |
| f__Neisseriaceae | | | 21.37 | 23.72 | 30.00 | 30.00 | -30.00 | -30.00 | 30.00 | 30.00 | - | -30.00 | |
| | g__ | | 30.00 | - | - | 27.49 | - | -30.00 | 30.00 | 30.00 | -30.00 | NA | NA |
| | | s__ | -14.00 | - | - | - | 16.98 | - | - | 18.73 | 16.01 | -17.77 | -15.72 |
| f__Pirellulaceae | | | 23.11 | -30.00 | -30.00 | -15.49 | -30.00 | -30.00 | -30.00 | - | 30.00 | 30.00 | |
| | g__ | | -17.21 | - | -13.14 | -13.67 | - | -30.00 | - | - | - | NA | NA |
| | | s__ | - | - | - | - | - | -23.61 | - | - | - | 29.92 | - |
| f__Propionibacteriaceae | | | -19.56 | 30.00 | 30.00 | 30.00 | -30.00 | -30.00 | - | - | 30.00 | -30.00 | |
| | g__Propioniba cterium | | -26.02 | - | 30.00 | 15.30 | -30.00 | -30.00 | - | - | 30.00 | NA | NA |
| f__Pseudomonadaceae | | | 30.00 | 30.00 | 30.00 | 30.00 | -30.00 | -30.00 | 22.97 | 22.87 | - | - | |
| | NA | | - | - | 30.00 | - | - | -30.00 | - | -25.74 | - | NA | NA |
| | | NA | - | - | - | - | - | -13.12 | - | - | - | 13.97 | 13.70 |
| f__Pseudomonadaceae | g__Pseudomo nas | | - | - | 18.28 | - | -30.00 | -30.00 | 30.00 | -30.00 | - | NA | NA |
| | | s__ | - | 8.61 | - | - | - | -12.72 | - | - | - | - | - |
| | g__ | | - | - | 30.00 | - | - | -30.00 | - | - | - | NA | NA |
| | | s__ | - | - | - | - | - | -11.43 | - | - | - | - | - |
| | | s__ | - | - | - | - | - | - | - | - | -19.63 | - | 26.47 |
| f__Rhodobacteraceae | | | 30.00 | 30.00 | 30.00 | 30.00 | -30.00 | 30.00 | 27.87 | 22.98 | - | -30.00 | |
| | g__Dinoroseo bacter | | -20.94 | 30.00 | 30.00 | -30.00 | -30.00 | 30.00 | - | - | - | NA | NA |
| | NA | | - | 30.00 | 30.00 | 30.00 | -30.00 | 30.00 | -28.29 | - | -28.38 | NA | NA |
| | | NA | 5.41 | 5.45 | - | - | -7.39 | - | - | - | - | - | - |
| | | NA | - | 10.03 | - | - | - | - | - | - | - | - | - |

| | | | | | | | | | | | | | |
|---|---|---|---|---|---|---|---|---|---|---|---|---|---|
| | | NA | 12.35 | 14.89 | 11.80 | - | -16.88 | -15.96 | -19.77 | - | - | - | 30.00 |
| | | NA | 7.08 | 7.30 | - | - | -10.05 | - | - | - | - | - | - |
| | | NA | 6.57 | 5.69 | 6.55 | - | - | - | - | - | - | - | - |
| | | NA | 15.13 | 13.88 | - | -11.72 | -27.14 | - | - | - | - | 30.00 | - |
| | | NA | 7.70 | 7.14 | 7.01 | - | - | - | - | - | - | - | - |
| | | NA | - | - | - | - | - | - | - | - | -21.50 | - | 23.72 |
| | | NA | 6.68 | 6.40 | - | - | - | - | -9.12 | - | -9.06 | - | 14.29 |
| | | NA | - | - | -21.46 | - | -17.37 | 22.62 | -15.92 | 28.24 | 17.18 | - | 22.33 |
| | g__Marivita | | - | 30.00 | 30.00 | -30.00 | -30.00 | 30.00 | - | - | -30.00 | NA | NA |
| | | s__ | - | 8.95 | - | - | - | - | - | - | - | - | - |
| | g__Shimia | | 21.07 | -25.77 | - | -15.17 | -30.00 | - | 30.00 | 30.00 | 30.00 | NA | NA |
| | | s__ | - | - | - | -16.85 | - | - | 16.53 | - | 17.60 | - | - |
| | g__Roseivivax | | - | - | 30.00 | - | - | -30.00 | 30.00 | - | - | NA | NA |
| | | s__ | 5.60 | 6.15 | - | - | -7.83 | - | - | - | - | - | - |
| | | s__ | - | - | - | -17.92 | - | - | - | - | - | - | - |
| | g__Anaerospora | | -30.00 | -20.26 | -30.00 | -30.00 | - | - | -25.12 | 30.00 | 25.00 | NA | NA |
| | g__Pseudoruegeria | | -27.30 | -24.06 | 28.58 | -22.38 | - | -30.00 | - | -30.00 | - | NA | NA |
| | | s__ | - | - | 10.20 | - | - | - | - | - | - | - | - |
| | g__ | | - | 30.00 | 30.00 | - | -30.00 | 30.00 | - | - | - | NA | NA |
| | | s__ | - | - | - | - | -22.34 | - | - | - | - | 21.75 | 19.24 |
| | | s__ | 6.70 | 6.79 | - | - | -11.84 | - | -9.28 | - | -9.34 | - | 15.12 |
| | | s__ | 9.51 | 8.95 | -12.58 | - | -14.80 | - | - | - | - | - | - |
| | g__Rhodovulum | | -30.00 | - | -27.92 | 30.00 | 30.00 | -26.19 | -23.96 | - | -30.00 | NA | NA |
| | | s__ | - | - | - | - | -11.80 | - | - | - | - | - | - |
| | | s__ | - | 11.01 | - | - | -29.01 | -11.52 | - | - | -19.79 | 24.17 | - |
| | | s__ | - | 5.49 | - | - | - | - | - | - | - | - | - |
| | g__Jannaschia | | -18.38 | - | - | 20.57 | -30.00 | -30.00 | 28.32 | 30.00 | 24.72 | NA | NA |

| | | | | | | | | | | | | | |
|---|---|---|---|---|---|---|---|---|---|---|---|---|---|
| o__Rhodospirillales | NA | NA | - | - | -18.54 | - | - | 18.35 | - | 14.51 | - | - | - |
| f__Sphingomonadaceae | | | - | - | - | -30.00 | - | -30.00 | - | -30.00 | - | 30.00 | |
| | g__Novosphingobium | | -16.79 | - | - | -30.00 | - | -30.00 | - | - | - | NA | NA |
| | | s__ | 11.48 | 11.43 | 11.96 | - | -20.80 | -14.67 | - | -16.20 | -17.79 | 26.73 | 30.00 |
| | | s__ | 11.10 | 11.00 | 11.88 | - | -20.13 | - | - | -16.64 | -17.47 | 27.10 | 30.00 |
| f__Staphylococcaceae | | | 30.00 | 30.00 | -30.00 | 30.00 | -30.00 | -30.00 | -30.00 | -30.00 | - | - | |
| | g__Staphylococcus | | - | - | -27.12 | 30.00 | - | -30.00 | -30.00 | - | - | NA | NA |
| | | s__ | - | - | - | - | - | - | - | - | - | - | 20.69 |
| | | s__ | - | - | - | - | - | - | - | - | - | - | 21.86 |
| f__Streptococcaceae | | | 30.00 | -30.00 | - | 30.00 | - | -30.00 | -30.00 | 30.00 | -30.00 | -30.00 | |
| | g__Streptococcus | | - | -26.70 | - | - | 27.88 | -30.00 | -30.00 | 30.00 | -30.00 | NA | NA |
| | | s__ | -12.68 | - | -12.80 | - | - | - | -28.42 | 30.00 | - | - | 30.00 |
| f__Synechococcaceae | | | 22.18 | 30.00 | 30.00 | - | -30.00 | -30.00 | -30.00 | 27.97 | 30.00 | -30.00 | |
| | g__Synechococcus | | - | - | 29.08 | - | -30.00 | -30.00 | -30.00 | - | 30.00 | NA | NA |
| f__Vibrionaceae | | | 30.00 | -30.00 | -30.00 | 30.00 | -30.00 | - | -30.00 | -30.00 | -30.00 | - | |
| | g__Vibrio | | - | -30.00 | -30.00 | 30.00 | - | - | -29.01 | -30.00 | -28.70 | NA | NA |
| | | s__ | - | - | -28.75 | - | -19.76 | 29.63 | - | - | - | 17.81 | 30.00 |
| o__Acidimicrobiales; f__wb1_P06 | | | - | - | -30.00 | 30.00 | -30.00 | -30.00 | 30.00 | -26.28 | 30.00 | 30.00 | |
| | g__ | | - | - | - | 30.00 | - | -30.00 | - | -30.00 | - | NA | NA |
| f__Xanthomonadaceae | | | 30.00 | 30.00 | - | 30.00 | -30.00 | -30.00 | - | 30.00 | - | - | |
| | g__Stenotrophomonas | | - | - | -24.05 | - | -24.97 | -30.00 | - | 27.36 | - | NA | NA |
| | | s__ | - | - | - | - | - | -10.54 | - | - | - | 17.19 | - |
| c: Alphaproteobactieria; NA | | | 28.84 | -30.00 | -30.00 | -18.68 | - | 29.95 | -30.00 | 30.00 | 30.00 | 30.00 | |
| | NA | | -16.77 | -13.23 | - | -11.87 | 27.57 | -26.58 | -30.00 | 26.51 | - | NA | NA |
| o__Rhodospirillales; NA | | | - | -30.00 | -16.67 | -30.00 | 29.21 | -30.00 | -30.00 | 28.63 | -28.40 | -30.00 | |
| | NA | | - | - | -26.08 | - | - | - | - | - | - | NA | NA |

**Supplementary Table S9. Resulting mapping file after filtering.**

OTUs were filtered from the dataset if they 1) failed to align with PyNAST to the GreenGenes database, 2) were annotated as mitochondrial or chloroplast sequences, or 3) had less than 100 counts across the entire dataset. Next, samples with less than 1000 reads were discarded (grey-colored sample rows).

| #SampleID | Tank | Nutrient | Temperature | Scarring | Colony | Treatment | Stress | Seq Depth |
|---|---|---|---|---|---|---|---|---|
| m.ch.15 | D | Control | Control | Control | C5 | Control | none | 12930 |
| m.ch.14 | D | Control | Control | Control | C4 | Control | none | 40274 |
| m.ch.11 | D | Control | Control | Control | C1 | Control | none | 73948 |
| m.ch.19 | L | Control | Control | Control | C9 | Control | none | 4 |
| m.ch.18 | L | Control | Control | Control | C8 | Control | none | 11320 |
| m.ch.17 | L | Control | Control | Control | C7 | Control | none | 24526 |
| m.ch.35 | F | Control | High T | Control | C5 | High T | single | 250 |
| m.ch.34 | F | Control | High T | Control | C4 | High T | single | 22203 |
| m.ch.31 | F | Control | High T | Control | C1 | High T | single | 64340 |
| m.ch.39 | G | Control | High T | Control | C9 | High T | single | 585 |
| m.ch.38 | G | Control | High T | Control | C8 | High T | single | 4562 |
| m.ch.37 | G | Control | High T | Control | C7 | High T | single | 7570 |
| m.ch.21 | F | Control | High T | Scarred | C4 | High T, scarred | double | 5 |
| m.ch.24 | F | Control | High T | Scarred | C4 | High T, scarred | double | 8192 |
| m.ch.25 | F | Control | High T | Scarred | C5 | High T, scarred | double | 17118 |
| m.ch.27 | G | Control | High T | Scarred | C7 | High T, scarred | double | 1501 |
| m.ch.28 | G | Control | High T | Scarred | C8 | High T, scarred | double | 3083 |
| m.ch.29 | G | Control | High T | Scarred | C9 | High T, scarred | double | 26644 |
| m.ch.95 | E | NH4+ | Control | Control | C5 | NH4+ | single | 4602 |
| m.ch.94 | E | NH4+ | Control | Control | C4 | NH4+ | single | 24321 |
| m.ch.91 | E | NH4+ | Control | Control | C1 | NH4+ | single | 52777 |
| m.ch.98 | K | NH4+ | Control | Control | C8 | NH4+ | single | 8199 |
| m.ch.99 | K | NH4+ | Control | Control | C9 | NH4+ | single | 21860 |
| m.ch.97 | K | NH4+ | Control | Control | C7 | NH4+ | single | 24625 |
| m.ch.111 | C | NH4+ | High T | Control | C1 | NH4+, High T | double | 86 |
| m.ch.115 | C | NH4+ | High T | Control | C5 | NH4+, High T | double | 2859 |
| m.ch.114 | C | NH4+ | High T | Control | C4 | NH4+, High T | double | 7614 |
| m.ch.117 | J | NH4+ | High T | Control | C7 | NH4+, High T | double | 1 |
| m.ch.118 | J | NH4+ | High T | Control | C8 | NH4+, High T | double | 2151 |
| m.ch.119 | J | NH4+ | High T | Control | C9 | NH4+, High T | double | 5455 |
| m.ch.105 | C | NH4+ | High T | Scarred | C5 | NH4+, High T, scarred | triple | 1616 |
| m.ch.101 | C | NH4+ | High T | Scarred | C1 | NH4+, High T, scarred | triple | 17469 |
| m.ch.104 | C | NH4+ | High T | Scarred | C4 | NH4+, High T, scarred | triple | 20733 |
| m.ch.107 | J | NH4+ | High T | Scarred | C7 | NH4+, High T, scarred | triple | 4 |
| m.ch.108 | J | NH4+ | High T | Scarred | C8 | NH4+, High T, scarred | triple | 1962 |

| | | | | | | | | |
|---|---|---|---|---|---|---|---|---|
| m.ch.109 | J | NH4+ | High T | Scarred | C9 | NH4+, High T, scarred | triple | 15391 |
| m.ch.84 | E | NH4+ | Control | Scarred | C4 | NH4+, scarred | double | 3058 |
| m.ch.85 | E | NH4+ | Control | Scarred | C5 | NH4+, scarred | double | 10134 |
| m.ch.81 | E | NH4+ | Control | Scarred | C1 | NH4+, scarred | double | 32454 |
| m.ch.89 | K | NH4+ | Control | Scarred | C9 | NH4+, scarred | double | 1691 |
| m.ch.88 | K | NH4+ | Control | Scarred | C8 | NH4+, scarred | double | 8458 |
| m.ch.87 | K | NH4+ | Control | Scarred | C7 | NH4+, scarred | double | 31459 |
| m.ch.54 | A | NO3- | Control | Control | C4 | NO3- | single | 8 |
| m.ch.55 | A | NO3- | Control | Control | C5 | NO3- | single | 4442 |
| m.ch.51 | A | NO3- | Control | Control | C1 | NO3- | single | 43207 |
| m.ch.57 | H | NO3- | Control | Control | C7 | NO3- | single | 13249 |
| m.ch.59 | H | NO3- | Control | Control | C9 | NO3- | single | 26058 |
| m.ch.58 | H | NO3- | Control | Control | C8 | NO3- | single | 27945 |
| m.ch.71 | B | NO3- | High T | Control | C1 | NO3-, High T | double | 0 |
| m.ch.74 | B | NO3- | High T | Control | C4 | NO3-, High T | double | 9623 |
| m.ch.75 | B | NO3- | High T | Control | C5 | NO3-, High T | double | 30273 |
| m.ch.79 | I | NO3- | High T | Control | C9 | NO3-, High T | double | 1070 |
| m.ch.77 | I | NO3- | High T | Control | C7 | NO3-, High T | double | 1139 |
| m.ch.78 | I | NO3- | High T | Control | C8 | NO3-, High T | double | 9360 |
| m.ch.61 | B | NO3- | High T | Scarred | C1 | NO3-, High T, scarred | triple | 5912 |
| m.ch.65 | B | NO3- | High T | Scarred | C5 | NO3-, High T, scarred | triple | 7723 |
| m.ch.64 | B | NO3- | High T | Scarred | C4 | NO3-, High T, scarred | triple | 10287 |
| m.ch.68 | I | NO3- | High T | Scarred | C8 | NO3-, High T, scarred | triple | 2 |
| m.ch.69 | I | NO3- | High T | Scarred | C9 | NO3-, High T, scarred | triple | 17725 |
| m.ch.67 | I | NO3- | High T | Scarred | C7 | NO3-, High T, scarred | triple | 23411 |
| m.ch.44 | A | NO3- | Control | Scarred | C4 | NO3-, scarred | double | 4904 |
| m.ch.45 | A | NO3- | Control | Scarred | C5 | NO3-, scarred | double | 21913 |
| m.ch.41 | A | NO3- | Control | Scarred | C1 | NO3-, scarred | double | 83601 |
| m.ch.47 | H | NO3- | Control | Scarred | C7 | NO3-, scarred | double | 9385 |
| m.ch.48 | H | NO3- | Control | Scarred | C8 | NO3-, scarred | double | 10564 |
| m.ch.49 | H | NO3- | Control | Scarred | C9 | NO3-, scarred | double | 15834 |
| m.ch.4 | D | Control | Control | Scarred | C4 | Scarred | single | 2334 |
| m.ch.1 | D | Control | Control | Scarred | C1 | Scarred | single | 5642 |
| m.ch.5 | D | Control | Control | Scarred | C5 | Scarred | single | 9368 |
| m.ch.9 | L | Control | Control | Scarred | C9 | Scarred | single | 3509 |
| m.ch.7 | L | Control | Control | Scarred | C7 | Scarred | single | 32292 |
| m.ch.8 | L | Control | Control | Scarred | C8 | Scarred | single | 59707 |
